# Daisy-chain gene drives for the alteration of local populations

**DOI:** 10.1073/pnas.1716358116

**Published:** 2019-04-02

**Authors:** Charleston Noble, John Min, Jason Olejarz, Joanna Buchthal, Alejandro Chavez, Andrea L. Smidler, Erika A. DeBenedictis, George M. Church, Martin A. Nowak, Kevin M. Esvelt

**Affiliations:** ^a^Program for Evolutionary Dynamics, Harvard University, Cambridge, MA 02138;; ^b^Department of Genetics, Harvard Medical School, Boston, MA 02115;; ^c^Media Laboratory, Massachusetts Institute of Technology, Cambridge, MA 02139;; ^d^Wyss Institute for Biologically Inspired Engineering, Harvard University, Cambridge, MA 02138;; ^e^Department of Pathology, Massachusetts General Hospital, Boston, MA 02114;; ^f^Department of Immunology and Infectious Diseases, Harvard School of Public Health, Boston, MA 02115;; ^g^Department of Mathematics, Harvard University, Cambridge, MA 02138;; ^h^Department of Organismic and Evolutionary Biology, Harvard University, Cambridge, MA 02138

**Keywords:** gene drive, CRISPR, evolutionary dynamics, evolutionary genetics, ecological engineering

## Abstract

If they are able to spread in wild populations, CRISPR-based gene-drive elements would provide new ways to address ecological problems by altering the traits of wild organisms, but the potential for uncontrolled spread tremendously complicates ethical development and use. Here, we detail a self-exhausting form of CRISPR-based drive system comprising genetic elements arranged in a daisy chain such that each drives the next. “Daisy-drive” systems can locally duplicate any effect achievable by using an equivalent self-propagating drive system, but their capacity to spread is limited by the successive loss of nondriving elements from one end of the chain. Releasing daisy-drive organisms constituting a small fraction of the local wild population can drive a useful genetic element nearly to local fixation for a wide range of fitness parameters without self-propagating spread. We additionally report numerous highly active guide RNA sequences sharing minimal homology that may enable evolutionarily stable daisy drive as well as self-propagating CRISPR-based gene drive. Especially when combined with threshold dependence, daisy drives could simplify decision-making and promote ethical use by enabling local communities to decide whether, when, and how to alter local ecosystems.

## Significance

CRISPR-based gene drive systems—genetic elements which could be engineered to rapidly spread traits through wild populations—could help solve some of humanity’s greatest ecological and public health problems. However, if released, current versions might spread through a nontarget population—possibly across political borders—greatly complicating decision-making. To address this issue, we describe a self-exhausting form of CRISPR-based gene drive called a “daisy-chain drive.” We develop mathematical models which suggest that daisy-chain-drive systems will not spread indefinitely through successive populations, and we report numerous CRISPR targeting sequences which could offer enhanced stability. Particularly if combined with threshold dependence, daisy-drive approaches may become a foundational technique for local ecological engineering.

**Fig. 1.**
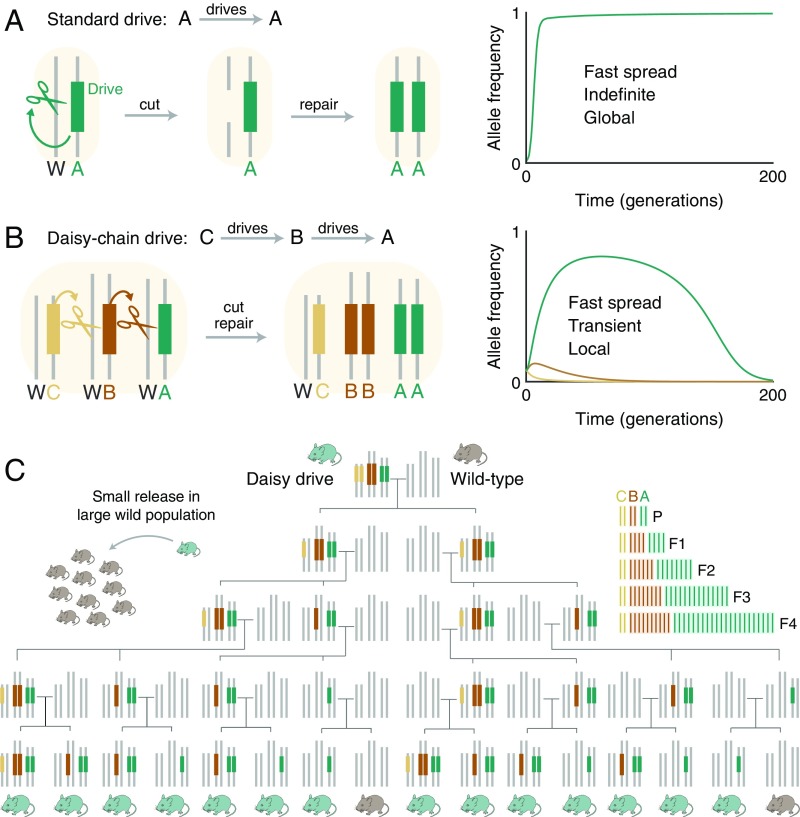
Comparison of self-propagating and daisy-chain gene drive. (*A*) Self-propagating CRISPR gene drives distort inheritance in a self-propagating manner by converting wild-type (W) alleles to drive alleles in heterozygous germline cells. (*B*) A daisy-drive system consists of a linear chain of serially dependent, unlinked drive elements; in this example, A, B, and C are on separate chromosomes. Elements at the base of the chain cannot drive and are successively lost over time via natural selection, limiting overall spread. (*C*) Family tree resulting from the release of a single daisy-drive organism in a resident wild-type population in the absence of selection. On the right is a graphical depiction of the total number of alleles per generation. Throughout, chromosome illustrations represent genotypes in germline cells.

RNA-guided gene-drive systems based on CRISPR nucleases raise the possibility that many types of genetic alterations could be spread through wild sexually reproducing species (1). These systems function by “homing,” or the conversion of heterozygotes to homozygotes in the germline, which renders offspring more likely to inherit the gene-drive element and the accompanying alteration than via normal Mendelian inheritance (2) (Fig. 1*A*). To date, gene-drive systems based on Cas9 have been demonstrated in yeast (3), fruit flies (4, 5), and two species of mosquito (6, 7). Suggested applications include eliminating vector-borne and parasitic diseases, promoting sustainable agriculture, and enabling ecological conservation by curtailing or removing invasive species (1).

The potential for self-propagating of standard RNA-guided gene-drive systems makes the technology attractive for attempts to address local ecological problems by gene manipulation, but the possibility of spread to additional populations of the target species (8, 9) tremendously complicates discussions of whether and how to proceed with any given intervention (10). While the development of resistance may prevent drive systems from reaching fixation in any given wild population, mathematical models predict that this does not prevent self-propagating alteration drives from spreading to most populations connected by gene flow. Population-suppression drives may behave similarly, given that suppression does not occur until the drive reaches high frequency, although explicit modeling is needed.

Technologies capable of unilaterally altering the shared environment require broad public support. Because people will not be able to opt out of technologies intended to alter the shared environment, ethical gene-drive research and development should be openly guided by the communities and nations that depend on the potentially affected ecosystems. Unfortunately, attaining this level of engagement becomes progressively more challenging as the size of the affected region increases. Candidate applications that will affect multiple nations could be delayed indefinitely due to a lack of agreement, particularly given the possibility that it may not be possible to conduct safely contained field trials (8, 9).

A method of preventing gene-drive systems from spreading indefinitely would greatly simplify community-directed development and deployment, while also enabling safe field testing. Existing theoretical self-exhausting strategies (11, 12) can locally spread cargo genes nearly to fixation if sufficiently many organisms (>30% of the local population) are released, while “threshold-dependent” drive systems, such as those using underdominance (13), will spread to fixation in small and geographically isolated subpopulations if organisms are released in an amount exceeding the threshold for population takeover (typically ≈50%). Toxin-based underdominance approaches are promising and have been demonstrated in fruit flies (14, 15), although they cannot directly suppress populations. All of these approaches involve releasing comparatively large numbers of organisms, which may not be politically, economically, or environmentally feasible for some applications.

A way to construct highly efficient yet locally confined RNA-guided drive systems could enable many potential applications for which neither self-propagating invasive drive systems nor existing local drives are suitable. Here, we describe “daisy drive,” a powerful yet self-exhausting form of local drive based on CRISPR-mediated homing in which the drive components are separated into an interdependent daisy chain. We additionally report guide RNA sequences required for evolutionary stability and safe use.

## Design and Modeling

A daisy-drive system consists of a linear series of genetic elements arranged such that each element drives the next in the chain (Fig. 1*B*). The final element in the chain, which carries the “cargo,” is driven to higher and higher frequencies in the population by the earlier elements in the chain. No element can drive itself (Fig. 1*C*). The bottom element is lost from the population over time, causing the next element to cease driving and be lost in turn. This process continues along the chain until, eventually, the population returns to its wild-type state (Fig. 1*B*).

The simplest form of daisy drive—a two-element chain—is obtained by separating CRISPR gene-drive components such that the cargo-carrying element, designated “A,” exhibits drive only in the presence of an unlinked, nondriving element, “B” (*SI Appendix*, Fig. S1). These “split drives” have been described (1), demonstrated (3), and recommended (16) as a stringent laboratory confinement strategy. Because any accidental release would involve only a small number of organisms carrying the B element, the driving effect experienced by the A element—and thus its spread—would be negligible in a large population (3). As long as the cargo confers a fitness cost to the host organism, both elements will eventually disappear due to natural selection.

We hypothesized that the spread of the cargo-carrying element, A, could be enhanced to useful levels by adding more elements to the base of the daisy chain. To explore this idea, we formulated a deterministic model which considers the evolution of a large population of diploid organisms affected by a daisy-drive system with elements spread across n loci (*SI Appendix*, sections 1 and 2). At each locus, there are three alleles: the wild-type (W), the corresponding daisy-drive element (D), and an allele which is resistant to the effects of the upstream daisy element (R). Such resistant alleles could exist before release in the form of standing genetic variation, or they could be created through misrepair following drive-mediated cleavage or by de novo mutation (5, 17, 18).

To model the effects of daisy drive in individuals, we made a few assumptions: (*i*) Daisy-drive alleles cut their targeted wild-type alleles with probability 1 (5, 6, 19); (*ii*) drive and resistant alleles are immune to drive-mediated cutting; and (*iii*) cutting is followed by homologous repair (HR) with probability H, leading to duplication of whatever allele is present at the homologous chromosome, or by nonhomologous end-joining (NHEJ) with probability 1−H, resulting in production of a new resistant allele.

**Fig. 2.**
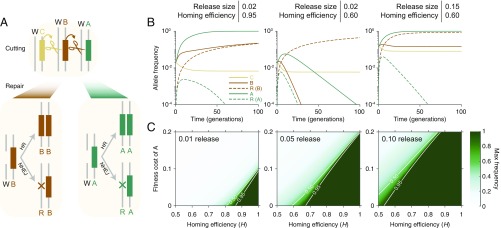
Dynamics of CBA daisy-chain gene-drive systems. (*A*) After being cut by an upstream daisy allele, a wild-type allele is repaired either by homologous recombination (HR), creating a second copy of the other allele at the locus, or by nonhomologous repair (e.g., NHEJ), leading to generation of a resistant allele. This process occurs in the germline and is independent at each locus. We assume that resistance at the cargo locus, A, is dominant lethal if inherited. (*B*) A highly efficient daisy drive (95% homing efficiency) with an 8% fitness cost for the cargo element seeded at 2% spreads the cargo nearly to fixation (*Left*). (*B*, *Center*) A low-efficiency drive (60%) with the same initial release size no longer allows drive spread. (*B*, *Right*) Increasing the release size of the inefficient drive to 15% again allows cargo spread to near fixation. (*C*) The maximum (Max) frequency achieved by cargo alleles as a function of the homing efficiency and the cargo fitness cost, for release sizes of 1% (*Left*), 5% (*Center*), and 10% (*Right*). Throughout, we assume a 0.01% fitness cost for C and B elements and neutral resistant alleles at the C and B loci.

The effect of a daisy-drive element at a particular locus (e.g., B) depends on the genotype at the next locus in the daisy chain (Fig. 2*A*). If that genotype is DD, DR, or RR, then no cutting occurs, and the genotype remains unchanged. If the genotype is WW, then both wild-type alleles are cut until the locus is converted to RR. Similarly, WR is converted to RR. However, if the genotype is WD, then the W allele is converted to D with probability H or to R with probability 1−H. We assume that standard Mendelian segregation occurs after conversion, so that, for example, individuals initially WD at a locus produce D gametes with probability (1+H)/2 or R gametes with probability (1−H)/2, assuming a daisy allele exists at the previous locus to facilitate the conversion. Finally, we assume that all loci undergo inheritance independently (i.e., all elements are unlinked, ideally on different chromosomes), so that the total probability of an individual producing a gamete of a particular haplotype is the product of its individual-locus inheritance probabilities. Details can be found in *SI Appendix*, section 2.2, with gamete-production probabilities explicitly written in *SI Appendix*, Eq. 7.

To model selection dynamics, we assumed that each daisy-drive element conferred a dominant fitness cost, $c_{i}$, on its host organism. Furthermore, we assumed that resistance at every upstream (noncargo) locus was neutral, while resistance at the cargo locus was dominant lethal. The latter requirement can be attained by targeting a haploinsufficient essential gene with the cargo element while including a genetically recoded copy in the drive construct (1, 17). All costs were assumed to be independent. (See *SI Appendix*, section 2.2 for further details. Fitness calculations are performed via *SI Appendix*, Eq. 6.)

While the requirement of dominant lethality for resistance at the cargo locus might seem prohibitively difficult to achieve, it is worth noting that recent experimental studies support the feasibility of this approach. In a study of CRISPR-Cas9 gene drive in yeast, DiCarlo et al. (3) constructed a drive targeting an essential gene, ABD1, while including a recoded copy in the drive construct, and no obvious impact on fitness was observed compared with wild-type strains. Furthermore, Ostrov et al. (20) used genetic recoding to successfully eliminate seven codons from 91% of essential genes in *Escherichia coli*, leading to an overall fitness cost of <10%. Models predict that cutting multiple sites within genes important for fitness is required for a drive system to affect an entire population (17, 21), and recent experiments featuring a two-gRNA drive element in fruit flies appear to provide evidence for simultaneous and reliable cutting by more than one gRNA (19).

Gene-drive dynamics are sensitive to homing efficiency (H) and fitness cost. In the four species examined, homing efficiency ranged from 37% to 99%, with almost all of the range stemming from variation in fruit flies. The rate was >99% for each of the many drive systems tested in yeast (3), 99.8% for the drive system in *Anopheles stephensi* (6), 87.3–99.7% for the three drive systems in *Anopheles gambiae* (7), and 37–95% for the three drive systems in the fruit fly, which varied with genetic background (4, 5). Fitness costs have not been rigorously measured, but costs associated with noncargo daisy-drive elements are expected to be much lower than typical cargoes (22, 23) because they will only encode guide RNAs. Potentially costly off-target cutting is minimal when using high-fidelity Cas9 variants (24, 25). If the target gene is haploinsufficient for proliferative gametogenesis, the cost may approach zero and the homing rate 100% in some species (*SI Appendix*, Fig. S2).

We studied a three-element daisy-drive system (CBA) via numerical simulation (Fig. 2). As expected, arbitrarily high frequencies of the cargo element, A, can be achieved by varying the release frequency. However, the system displayed high sensitivity to the homing rate and cargo cost. In particular, moderate release sizes (>10% of the resident population) were required to drive costly cargoes if homing efficiency was on the lower end of observed drive systems (≈60%).

**Fig. 3.**
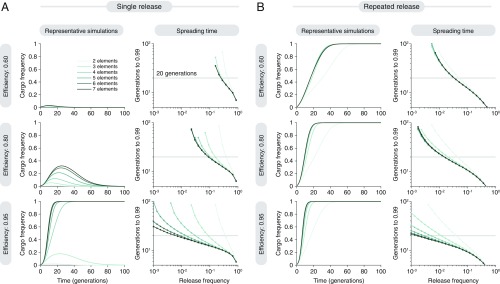
Quantitative evaluation of cargo spread in a single population, for single and repeated releases. (*A*) Results assuming a single release of daisy-drive organisms in a wild population. (*A*, *Left*) Representative simulations assuming a 1% release. (*A*, *Right*) Time to achieve 99% frequency for varying release frequency. (*B*) Results assuming a constant rate of release of daisy-drive organisms. (*B*, *Left*) Representative simulations, assuming an initial 1% release with a subsequent release rate of 0.01 per generation (see *SI Appendix*, section 2.3 for details). (*B*, *Right*) Time to 99% frequency with varying release rate, which we set as both the initial release frequency as well as the subsequent continuous release frequency, indicated by the horizontal axis. (See *SI Appendix*, section 2.3 for details on continuous release.) All simulations assume a 10% cargo cost, 0.01% cost per upstream element, and 60% (*A* and *B*, *Top*), 80% (*A* and *B*, *Middle*), or 95% (*A* and *B*, *Bottom*) homing efficiencies.

We next explored the effects of adding additional elements to the daisy-drive system as a potential means of increasing potency. We observed that longer chains led to much stronger drive (Fig. 3*A*). At a homing efficiency of 95% per daisy-drive element, six- and seven-element systems driving a cargo with a 10% cost could be released at frequencies as low as 1% and still have >99% frequency in <20 generations. On a per-organism basis, these were 10- to 1,000-fold more efficient than simply releasing organisms with the cargo, depending on the homing efficiency (*SI Appendix*, Fig. S3).

Adjusting the model to include repeated releases in every subsequent generation, we observed that daisy drives can readily alter local populations if repeatedly released in very small numbers, although the benefit of repeated release was lost when the repeated release size became large (>5%) (Fig. 3*B*). This may be useful for applications that must affect large geographic regions over extended periods of time, as well as for local eradication campaigns (26). (More accurately, we simulated a continuous release of engineered individuals into a wild population for convenience in doing the simulations; see *SI Appendix*, section 2.3 for details on this implementation.)

Given that the cargo element could achieve arbitrarily high frequencies in a population, we next asked how long the cargo might persist after attaining a high frequency. Thorough quantitative analysis of this point will be an important direction for future work, but as a first step, we here sought to understand qualitatively how each of our model parameters impacts this persistence time. To accomplish this, we returned to our basic three-element (CBA) model and performed the following procedure: (*i*) We chose a particular set of parameter values such that the drive could attain at least 50% frequency across a range of nearby values for each parameter. (*ii*) We then varied each parameter individually, while measuring the number of generations that the cargo element remained >50% frequency, thus isolating the effect of each parameter.

The results of this analysis are shown in *SI Appendix*, Fig. S4. Overall, we found that the persistence time (i.e., the number of generations >50% frequency) varied significantly across plausible ranges for the parameters in our model. The most dramatic effect was observed by varying the fitness cost of resistance at the cargo element, s. We found that, roughly, if s was less than c, the fitness cost of the cargo element, then the cargo was unlikely to achieve near-fixation, while if s>c, then resistance at the cargo element was more deleterious than the cargo itself, and the cargo could remain in the population indefinitely, barring mutations that inactivate its function. Regarding the other parameters, we found that the persistence time was inversely proportional to c and more robust to small perturbations in the homing efficiency, H, release frequency, and fitness cost, d, associated with upstream elements (C, B).

Finally, we considered the potential for daisy-drive systems to affect local populations of invasive species on islands or other regions with limited gene flow. To study the extent of spread between populations, we formulated a metapopulation model consisting of N populations connected by pairwise gene-flow rates in a directed-graph-based structure (*SI Appendix*, sections 3 and 4). Within each population, we assumed random mating with selection and germline dynamics identical to those described in the single-population model above.

To begin our analysis of this model, we studied a particular case consisting of five equally sized populations connected in a chain, with each population exchanging individuals with its neighboring populations immediately before and/or after it in the chain (*SI Appendix*, section 5). We further assumed gene-flow rates of $10^{-2}$ between each pair of neighboring populations.

**Fig. 4.**
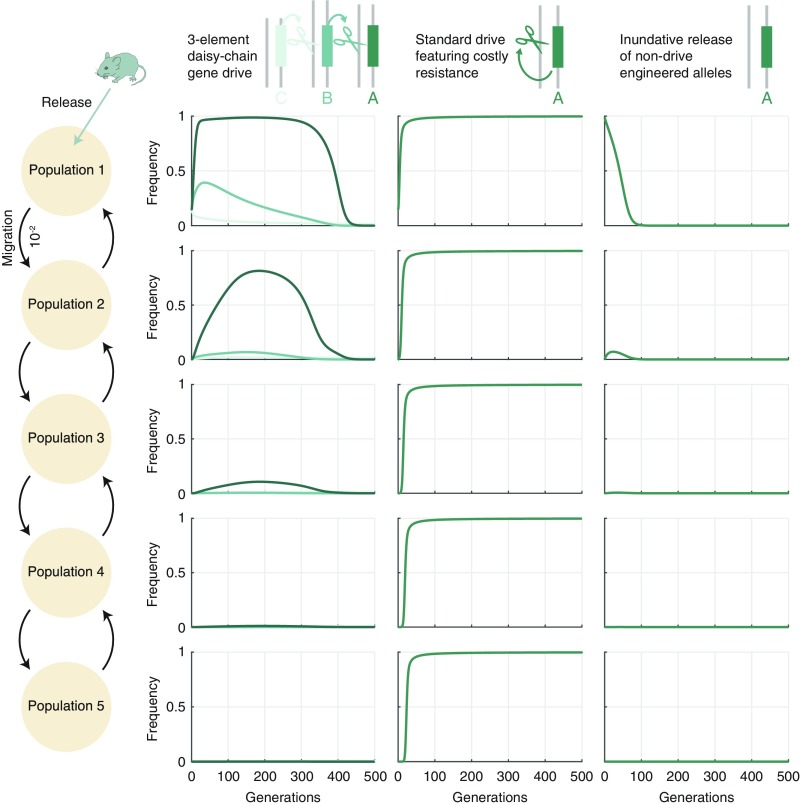
Modeling daisy-drive containment in a system of populations connected by gene flow. (*Left*) Illustration of the population structure: Five populations with equal sizes are connected in a chain, and each neighboring pair has bidirectional gene flow with rate $10^{-2}$ in each direction. The three columns in *Right* then correspond to the three scenarios described in the text: CBA daisy-chain drive (first column), self-propagating (“standard”) drive with multiple gRNAs targeting an essential gene, as in ref. 17 (second column), and nondrive inundative release (third column). Frequencies over time are indicated for each allele in each of the populations. Drive-based simulations (daisy chain and standard) assume 80% homing efficiency, 10% dominant cargo element fitness cost, and 15% release frequency. Daisy-chain drive simulations further assume 0.01% upstream element (C, B) fitness cost. Inundative release simulations assume 10% dominant fitness cost and 99.9% release. See *SI Appendix*, section 5 for details.

Given this population structure, we compared three scenarios, each beginning with a release of engineered individuals in the population at the beginning of the chain (Fig. 4): (*i*) a three-element (CBA) daisy-chain drive; (*ii*) a standard self-propagating drive element designed with multiple gRNAs to mitigate resistance (adapting the model from ref. 17; see *SI Appendix*, section 5.2 for details); and (*iii*) an inundative release of engineered alleles which do not drive at all. (This scenario was simulated by using the same model as in scenario 2, as described in *SI Appendix*, section 5.2, except that we set the cutting rate to zero so that standard Mendelian inheritance occurs. Specifically, we set q=0 in the inheritance probability equations of SI section 7.3.2 of ref. 17.)

To ensure that the three scenarios were comparable, we used identical parameters where applicable. In the two drive scenarios (1 and 2), we assumed a moderate 80% homing efficiency, 15% release size, and 10% fitness cost for the cargo element (as well as perfect cutting efficiency, as described above). Additionally, for daisy drive, we continued assuming a low fitness cost for the C and B elements (0.01%). For the inundative release scenario, we assumed an identical 10% dominant fitness cost for the engineered element, but we set the release size to 99.9%.

Results for these three initial scenarios can be found in Fig. 4. For the daisy-chain drive, we found that the cargo element could be driven to near-fixation in its initial-release population, while attaining significant frequency (≈0.8) in the second population, low frequency in the third population (≈0.2), and only negligible frequencies in the subsequent populations. Moreover, transience of the cargo element is ensured in the initial population by an influx of wild-type individuals. This constitutes a mechanism for transience which cannot be captured by our single-population model; as such, we would expect our persistence time results discussed above and presented in *SI Appendix*, Fig. S4 to be substantially different in this more realistic multiple-population context. In contrast, the self-propagating drive rapidly spread to near-fixation in all populations.

We then further analyzed interpopulation spread in this model via numerical simulation, and additional results can be found in *SI Appendix*, Fig. S5. Specifically, we varied the migration rate between $10^{-4}$ and $10^{-1}$ for each of the three scenarios described above and measured the maximum frequency achieved by each allele across 500-generation simulations. We found that, for migration rates <$10^{-2}$ (the value assumed in Fig. 4), maximum daisy-chain cargo frequency in the second population decreased roughly linearly with the migration rate, whereas self-propagating drive approached fixation in all populations, even for very low migration rates in the absence of resistance. Notably, the resistant allele at the B locus can exhibit high frequencies in multiple populations due to its assumed low fitness cost; however, this effect could potentially be mitigated by engineering that element to select against resistance in the same way as the A element.

## Evolutionary Stability and CRISPR Multiplexing

**Fig. 5.**
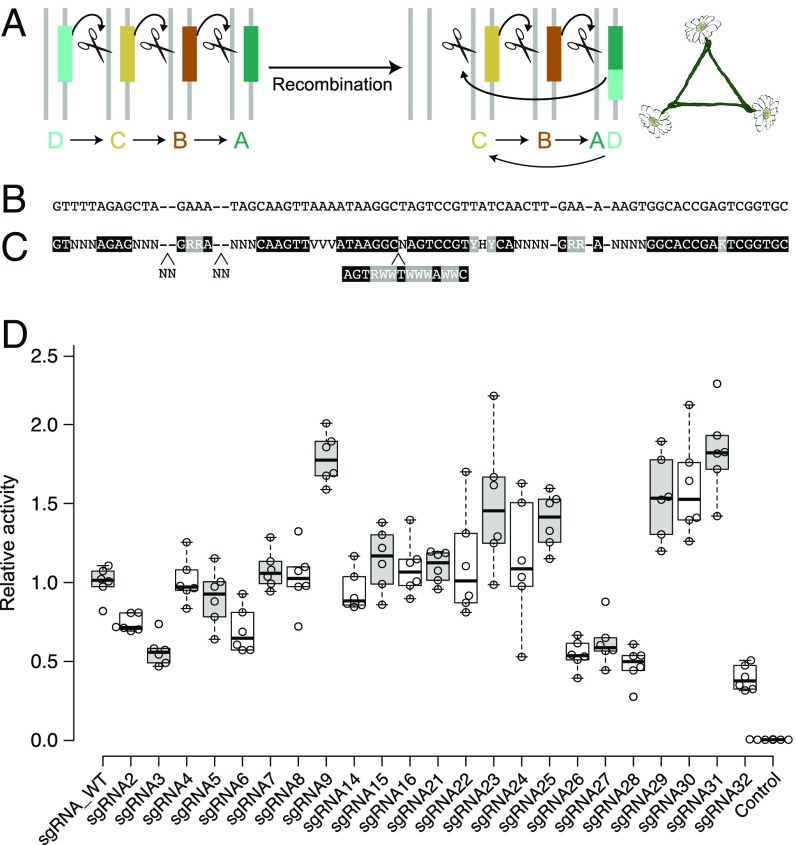
Preventing the formation of “daisy necklaces.” (*A*) Any recombination event that moves a DNA template for guide RNA from one element to another, where it will be reliably copied, could create a daisy necklace capable of self-propagating drive. (*B*) Because promoters can be changed, repetition of the conserved DNA template sequence is a key problem. (*C*) Using existing data, we generated a template identifying candidate positions presumed tolerant of sequence changes. (*D*) Relative activities of candidate guide RNAs generated from the template were assayed by using a Cas9 transcriptional activator screen using a tdTomato reporter in human cells.

Despite these promising theoretical results, current technological limitations preclude the safe use of daisy-drive systems. Specifically, a recombination event that moves one or more guide RNAs within an upstream element of the chain into any downstream element could convert a linear daisy-drive chain into a “necklace” analogous to self-propagating gene-drive systems anticipated to spread to populations worldwide (Fig. 5*A*).

One way to reliably prevent such events is to eliminate regions of homology between the elements. Promoter homology can be removed by using different U6, H1, or tRNA promoters to express the required guide RNAs (27–29); if there are insufficient promoters, then each can drive expression of multiple guide RNAs by using tRNA (30, 31) or miRNA processing (32–34). However, each element must still encode multiple guide RNAs >80 base pairs in length to prevent the creation of drive-resistant alleles, precluding safe and stable daisy-drive designs.

One alternative is to use a distinct orthogonal CRISPR system for every daisy element (35) (*SI Appendix*, Fig. S6). Unfortunately, it is more difficult to find multiple promoters suitable for nuclease expression than for guide RNA expression, and the fitness cost is likely higher than an equivalent guide RNA element. We accordingly sought to identify highly active guide RNA sequences for *Streptococcus pyogenes* Cas9 with minimal homology to one another that could enable safe daisy drive using only a single CRISPR nuclease.

We compared known tracrRNA, crRNA, and alternative sgRNA sequences for CRISPR systems related to that of *S. pyogenes* to identify bases tolerant of variation within the sequence of the most commonly used sgRNA (Fig. 5 *B* and *C*). We then created dozens of sgRNA variants designed to be as divergent from one another as possible. Assaying these by using a sensitive tdTomato-based transcriptional activation reporter in human cell culture identified 15 different sgRNAs with activities comparable to the self-propagating version (Fig. 5*D*). Activity increased with the length of the first stem, in agreement with other reports (36) (*SI Appendix*, Figs. S7 and S8). This set of minimally homologous sgRNAs can be used to construct stable daisy-drive systems of up to five elements with four sgRNAs per driving element and will also facilitate multiplexed Cas9 targeting in the laboratory by permitting the commercial synthesis of DNA fragments encoding many sequence-divergent guide RNAs. Future studies will need to examine the stability of the resulting daisy-drive systems in large populations of animal models.

Importantly, our divergent guide RNAs will also enable self-propagating CRISPR gene-drive elements to overcome the problem of instability caused by including multiple repetitive guide RNA sequences in the drive cassette (37), which is needed to overcome drive-resistant alleles (17, 21). Using nonrepetitive guides may consequently allow stable and efficient self-propagating drive systems to affect every organism in the target population.

## Construction and Deployment

On a practical level, researchers need only construct one “generic” daisy drive strain per species that could subsequently be loaded with any desired cargo. This generic daisy-drive system, which would typically harbor the nuclease gene in the A position, could be used in three different ways.

First, one or more “effector” elements carrying cargo genes and guide RNAs sufficient to drive themselves in the presence of nuclease could be added directly to the generic daisy-drive strain. In this configuration, the nuclease-encoding element would become the B element with the effector(s) in the A position. These daisy-drive organisms would then be mass-produced and released in a single-strain, single-stage approach.

Second, the generic daisy-drive strain could be released in the target region alongside organisms carrying effector elements already present from releases in adjacent areas. Matings in the wild would then combine the daisy-chain and effector elements, allowing more precise control in spreading the effector cargo into new areas.

Third, the generic daisy-drive strain could be released without an effector, and the spread of the nuclease gene could be monitored. This would allow for precise prediction and tuning of the region affected before a later release of strains carrying effector elements to initiate the desired effect. If necessary, the extent of nuclease spread could be adjusted by releasing wild-type or more daisy-drive organisms to fine-tune the areas affected, allowing a level of control not afforded by classic gene-drive architectures, albeit one that is imperfect due to stochastic migration. Superior control might be obtained by coupling daisy drive to underdominance to limit dispersion of the alteration to areas in which it is already in the majority (38).

## Field Trials and Safeguards

Ecological problems such as malaria are so widely distributed geographically that addressing them may require self-propagating CRISPR-based gene-drive systems. However, alteration drive systems of this type arguably cannot be tested in field trials without a substantial risk of eventual international spread (8, 9), and future models may demonstrate that the same is true of self-propagating suppression drive. While some might argue that the only relevant test is an empirical study in large wild populations, a positive result in that experiment would by definition lead to unauthorized international spread absent a diplomatic agreement in advance of the trial. Daisy-drive systems, which are capable of mimicking the molecular effects of any self-propagating drive on a local level, may offer a potential solution.

Notably, daisy-drive systems might be used to directly suppress target populations by imposing a genetic load or by sex-biasing the local population, exactly as would equivalent self-propagating CRISPR-based drive systems. For example, a daisy drive that disrupts female fertility genes, such as those recently identified in malarial mosquitoes (7), might encode the basal element of the daisy chain on the Y chromosome or an equivalent male-specific locus, thereby ensuring that most male offspring preferentially inherit the complete daisy suppression drive system and enabling out-crossing to wild females during production (*SI Appendix*, Fig. S9). As with a Y-linked suppression element (39), such males should suffer no direct fitness costs from the genetic load relative to competing wild-type males.

Finally, scientists currently have few attractive options for controlling unauthorized or accidentally released CRISPR-based gene-drive systems. While it is possible to overwrite genome-level alterations and undo phenotypic changes by using immunizing reversal drives (1), these countermeasures must necessarily spread to the entire population to immunize them against the unwanted drive system; strategies based on pure reversal drives (3) or variations such as gene-drive “brakes” (40) should only slow it down. In contrast, daisy-drive systems may be powerful enough to eliminate all copies of an unwanted self-propagating drive system via local immunizing reversal, population suppression, or both (conceptually illustrated in *SI Appendix*, Fig. S10). Feasibility, especially in species with high dispersal rates, should be investigated by modeling and metapopulation experiments.

## Conclusions

RNA-guided gene drives based on CRISPR have generated considerable excitement as a potential means of addressing otherwise intractable ecological problems. While experiments have raced ahead, the potential for international spread once released into a wild population may prove a formidable barrier, given the need for public support and international regulatory approval, which may not be achievable if the proposed system cannot be safely tested in the field. These ethical and diplomatic complications are most acute for drive systems aiming to solve the most urgent humanitarian problems, including malaria, schistosomiasis, dengue, and other vector-borne and parasitic diseases, as the lack of international agreement could delay releases by years or even decades.

Similarly, the potential for RNA-guided drive systems to be released accidentally or unilaterally has led to many calls for caution and expressions of alarm, not least from scientists in the vanguard of the field (1, 16, 41). Any such event could have potentially devastating consequences for public trust and support for future interventions.

In contrast, our results suggest that daisy-drive systems could be safely developed in the laboratory, assessed in the field, and deployed to accomplish transient alterations that should minimally impact other nations or jurisdictions. They might be used to locally duplicate the effects of a self-propagating drive system for safe field studies, to efficiently alter entire local populations with limited gene flow such as those on islands, or to accomplish transient changes to pockets of mainland populations.

However, it is essential to note that daisy drive alone cannot prevent the spread of engineered genes into adjacent populations across political boundaries (42). Addressing this problem will require triggering a threshold-dependent drive system after the daisy drive has been exhausted to actively eliminate engineered alleles from adjacent populations where they are in the minority (38).

By using molecular constraints to limit generational and geographic spread in a tunable manner, daisy-drive approaches could expand the scope of ecological engineering by enabling local communities to make decisions concerning their own local environments.

## Materials and Methods

### Guide RNA Design.

We examined existing data on guide RNA variants and corresponding activities as well as the crystal structure of *S. pyogenes* Cas9 in complex with sgRNA to identify bases that would likely tolerate mutation. Using this information, we constructed a set of 20 sgRNAs and assayed activity (see below) using only two replicates to identify sequence changes that were harmful to activity. These experiments suggested that the large insertion found in sgRNAs from closely related bacteria was well-tolerated in only one case. It was consequently removed, and additional sgRNAs were designed. All candidates were then assayed to identify those with sufficiently high activity. Future experiments requiring additional highly divergent sgRNAs, such as daisy suppression drives in which the A element encodes many guide RNAs that disrupt multiple recessive fertility genes at multiple sites, will require a more comprehensive, library-based approach to activity profiling.

### Measuring Guide RNA Activity.

HEK293T cells were grown in DMEM (Life Technologies) fortified with 10% FBS (Life Technologies) and penicillin/streptomycin (Life Technologies). Cells were incubated at a constant temperature of 37°C with 5% CO_2_. In preparation for transfection, cells were split into 24-well plates, divided into ∼50,000 cells per well. Cells were transfected by using 2 μL of Lipofectamine 2000 (Life Technologies) with 200 ng of dCas9 activator plasmid, 25 ng of guide RNA plasmid, 60 ng of reporter plasmid, and 25 ng of EBFP2-expressing plasmid.

Fluorescent transcriptional activation reporter assays were performed by using a modified version of Addgene plasmid no. 47320, a reporter expressing a tdTomato fluorescent protein adapted to contain an additional gRNA binding site 100 bp upstream of the original site. gRNAs were cotransfected with reporter, dCas9-VPR, a tripartite transcriptional activator fused to the C terminus of nuclease-null *S. pyogenes* Cas9, and an EBFP2-expressing control plasmid into HEK293T cells. At 48 h posttransfection, cells were analyzed by flow cytometry. To exclusively analyze transfected cells, cells with <$10^{3}$ arbitrary units of EBFP2 fluorescence were ignored. The preliminary screen of the initial 20 designs was performed with only two replicates to identify critical bases. Experiments evaluating the final set of sgRNA sequences were performed with six biological replicates.

### Code Availability.

All code was custom-written in Matlab and is available. An interactive, web-based simulation application can be found at daisydrives.media.mit.edu, with source code at github.com/erika-alden/bokeh_daisy_drive.

## Supplementary Material

Supplementary File
