# Supplementary material for: Daisy-chain gene drives for the alteration of local populations
Source: Proc Natl Acad Sci U S A. 2019 Apr 2;116(17):8275–82. doi: 10.1073/pnas.1716358116 (PMC6486765; doi:10.1073/pnas.1716358116)
Supplement: Supplementary File [file pnas.1716358116.sapp.pdf]

PNAS
www.pnas.org

Supplementary Information for

Daisy-chain gene drives for the alteration of local populations

Charleston Noble, John Min, Jason Olejarz, Joanna Buchthal, Alejandro Chavez, Andrea L. Smidler, Erika A. DeBenedictis, George M. Church, Martin A. Nowak and Kevin M. Esvelt

Kevin Esvelt and George Church.
Email: esvelt@mit.edu (K.M.E.), gmc@harvard.edu (G.M.C.)

This PDF file includes:

Supplementary text
Figs. S1 to S11
References for SI reference citations
Correspondence with the Editorial Board

www.pnas.org/cgi/doi/10.1073/pnas.1716358116

Supplementary Information Text

1 Evolutionary dynamics of a daisy drive construct

We first describe a daisy drive system consisting of only two elements. This simple case demonstrates the principle behind daisy drive engineering. We then describe a daisy drive system with an arbitrary number of elements.

1.1 Model for the evolutionary dynamics of a 2-element daisy drive

We consider a wild population of diploid organisms and focus on two loci, "1" and "2". The wild-type alleles at the two loci are 1_W and 2_W, and we denote by $1_{WW}2_{WW}$ the genotype of an individual that is homozygous for both. Using CRISPR genome editing technology, one can engineer what we refer to as "daisy" alleles at both loci (1_D and 2_D). They function as follows. The 1_D allele effects cutting of the 2_W allele in an individual's germline. We assume that the two loci are independent and that a single copy of 1_D always induces cutting of the 2_W allele. If an individual has genotype $1_{WD}2_{WW}$ or $1_{DD}2_{WW}$, then the drive allele at the first locus cuts and disrupts both wild-type alleles at the second locus, and the resulting gametes are nonviable. If an individual has genotype $1_{WD}2_{WD}$ or $1_{DD}2_{WD}$, then the drive allele at the first locus cuts the wild-type allele at the second locus, and one of two things can happen. If a homing event occurs, then the drive allele at the second locus is successfully copied into the position of the shredded wild-type allele, resulting in gametes that necessarily have the drive allele at the second locus. If a homing event does not occur, then the resulting gametes are nonviable. This results in super-Mendelian inheritance of the 2_D allele in a 1_D-mediated fashion. Importantly, the 1_D allele undergoes standard inheritance and does not facilitate its own spread similarly.

(Notice that in this simplified treatment, we do not explicitly study evolution with a resistant allele, as described in the main text. This simplified model illustrates the principle behind daisy drive engineering without concern for complications arising from emergence of resistance. In Section 2 of this Supplementary Information, we introduce resistance into the model.)

To see how the daisy drive works, consider Table 1, which is understood as follows:

Genotype	1_W2_W	1_W2_D	1_D2_W	1_D2_D
$1_{WW}2_{WW}$	1	0	0	0
$1_{WW}2_{WD}$	$\frac{1}{2}F$	$\frac{1}{2}F$	0	0
$1_{WW}2_{DD}$	0	F	0	0
$1_{WD}2_{WW}$	0	0	0	0
$1_{WD}2_{WD}$	0	$\frac{1}{2}HF$	0	$\frac{1}{2}HF$
$1_{WD}2_{DD}$	0	$\frac{1}{2}F$	0	$\frac{1}{2}F$
$1_{DD}2_{WW}$	0	0	0	0
$1_{DD}2_{WD}$	0	0	0	HF
$1_{DD}2_{DD}$	0	0	0	F

Table 1: Gamete production table showing the relative rates at which individuals of each genotype (rows) produce gametes of each haplotype (columns).

Gametes of haplotype 1_W2_W are produced in the following ways:

- $1_{WW}2_{WW}$ individuals produce only $1_W 2_W$ gametes. We set the rate of production of $1_W 2_W$ gametes by $1_{WW}2_{WW}$ individuals to be 1.
- $1_{WW}2_{WD}$ individuals produce gametes with a wild-type allele at the second locus with probability 1/2. There is a fitness effect, F, due to the payload of the drive allele at the second locus. So $1_{WW}2_{WD}$ individuals produce $1_W 2_W$ gametes at relative rate $F/2$.

Gametes of haplotype $1_W 2_D$ are produced in the following ways:

- $1_{WW}2_{WD}$ individuals produce gametes with a drive allele at the second locus with probability 1/2. There is a fitness effect, F, due to the payload of the drive allele at the second locus. So $1_{WW}2_{WD}$ individuals produce $1_W 2_D$ gametes at relative rate $F/2$.
- $1_{WW}2_{DD}$ individuals produce only $1_W 2_D$ gametes. There is a fitness effect, F, due to the payload of the drive allele at the second locus. So $1_{WW}2_{DD}$ individuals produce $1_W 2_D$ gametes at relative rate F.
- $1_{WD}2_{WD}$ individuals produce gametes with a wild-type allele at the first locus with probability 1/2. The action of the drive allele at the first locus is to cut the wild-type allele at the second locus, and homing occurs with probability H. There is a fitness effect, F, due to the payload of the drive allele at the second locus. So $1_{WD}2_{WD}$ individuals produce $1_W 2_D$ gametes at relative rate $HF/2$.
- $1_{WD}2_{DD}$ individuals produce gametes with a wild-type allele at the first locus with probability 1/2. There is a fitness effect, F, due to the payload of the drive allele at the second locus. So $1_{WD}2_{DD}$ individuals produce $1_W 2_D$ gametes at relative rate $F/2$.

Gametes of haplotype $1_D 2_D$ are produced in the following ways:

- $1_{WD}2_{WD}$ individuals produce gametes with a drive allele at the first locus with probability 1/2. The action of the drive allele at the first locus is to cut the wild-type allele at the second locus, and homing occurs with probability H. There is a fitness effect, F, due to the payload of the drive allele at the second locus. So $1_{WD}2_{WD}$ individuals produce $1_D 2_D$ gametes at relative rate $HF/2$.
- $1_{WD}2_{DD}$ individuals produce gametes with a drive allele at the first locus with probability 1/2. There is a fitness effect, F, due to the payload of the drive allele at the second locus. So $1_{WD}2_{DD}$ individuals produce $1_D 2_D$ gametes at relative rate $F/2$.
- $1_{DD}2_{WD}$ individuals have only the drive allele at the first locus. The action of the drive allele at the first locus is to cut the wild-type allele at the second locus, and homing occurs with probability H. There is a fitness effect, F, due to the payload of the drive allele at the second locus. So $1_{DD}2_{WD}$ individuals produce $1_D 2_D$ gametes at relative rate HF.
- $1_{DD}2_{DD}$ individuals produce only $1_D 2_D$ gametes. There is a fitness effect, F, due to the payload of the drive allele at the second locus. So $1_{DD}2_{DD}$ individuals produce $1_D 2_D$ gametes at relative rate F.

(Notice that if H is interpreted as the homing probability and F is interpreted as the fitness effect due to the drive payload, then Table 1 is naturally interpreted as describing drive that occurs in the embryo. That is, individuals with at least one copy of the drive allele at the first locus and a single copy of the drive allele at the second locus shred the wild-type allele at the second locus during

embryonic development. And if homing does not occur, then the resulting, mature individuals are nonviable since the W (or D) allele is haploinsufficient. But Table 1 also effectively describes the production of gametes in the case of meiotic drive. The subtle distinction in that case would be that, if cutting occurs and homing does not follow, then $1_{WD}2_{WD}$ and $1_{DD}2_{WD}$ individuals produce a nonzero amount of gametes with a mutilated wild-type allele at the second locus. But when those gametes pair with any other gamete, the resulting individuals are necessarily nonviable, and so, effectively, $1_{WD}2_{WD}$ and $1_{DD}2_{WD}$ individuals only produce gametes with a drive allele at the second locus.)

Using these rules, we can formally express the rates at which the four types of gametes are produced in the population. We denote by $g(z)$ the rate (with implicit time-dependence) at which gametes with haplotype z are produced by individuals in the population.

$$
\begin{aligned}
g(1_W 2_W) &= x(1_{WW}2_{WW}) + \frac{1}{2}Fx(1_{WW}2_{WD}) \\
g(1_W 2_D) &= \frac{1}{2}Fx(1_{WW}2_{WD}) + Fx(1_{WW}2_{DD}) + \frac{1}{2}HFx(1_{WD}2_{WD}) + \frac{1}{2}Fx(1_{WD}2_{DD}) \\
g(1_D 2_W) &= 0 \\
g(1_D 2_D) &= \frac{1}{2}HFx(1_{WD}2_{WD}) + \frac{1}{2}Fx(1_{WD}2_{DD}) + HFx(1_{DD}2_{WD}) + Fx(1_{DD}2_{DD})
\end{aligned}
$$

Here, $x(z)$ is the frequency of individuals with genotype z.

The selection dynamics are then modeled by the following system of equations:

$$
\begin{aligned}
\dot{x}(1_{WW}2_{WW}) &= g(1_W 2_W)^2 - \psi^2 x(1_{WW}2_{WW}) \\
\dot{x}(1_{WW}2_{WD}) &= 2g(1_W 2_W)g(1_W 2_D) - \psi^2 x(1_{WW}2_{WD}) \\
\dot{x}(1_{WW}2_{DD}) &= g(1_W 2_D)^2 - \psi^2 x(1_{WW}2_{DD}) \\
\dot{x}(1_{WD}2_{WW}) &= 2g(1_W 2_W)g(1_D 2_W) - \psi^2 x(1_{WD}2_{WW}) \\
\dot{x}(1_{WD}2_{WD}) &= 2g(1_W 2_D)g(1_D 2_W) + 2g(1_W 2_W)g(1_D 2_D) - \psi^2 x(1_{WD}2_{WD}) \\
\dot{x}(1_{WD}2_{DD}) &= 2g(1_W 2_D)g(1_D 2_D) - \psi^2 x(1_{WD}2_{DD}) \\
\dot{x}(1_{DD}2_{WW}) &= g(1_D 2_W)^2 - \psi^2 x(1_{DD}2_{WW}) \\
\dot{x}(1_{DD}2_{WD}) &= 2g(1_D 2_W)g(1_D 2_D) - \psi^2 x(1_{DD}2_{WD}) \\
\dot{x}(1_{DD}2_{DD}) &= g(1_D 2_D)^2 - \psi^2 x(1_{DD}2_{DD})
\end{aligned}
$$

Here, an overdot denotes the time derivative, d/dt. Throughout this Supplementary Information, we omit explicitly writing the time dependence of our dynamical quantities. Note that this formulation assumes random mating, i.e., that two random gametes come together to form an individual. Also note that products $g(y)g(z)$ represent the pairings of different gametes. At any given time, we require that the total number of individuals sums to one:

$$\sum_z x(z) = 1$$

To enforce this density constraint, we set

$$\psi = g(1_W 2_W) + g(1_W 2_D) + g(1_D 2_W) + g(1_D 2_D)$$

1.2 Model for the evolutionary dynamics of an n-element daisy drive

We can apply the same engineering to a daisy drive chain of arbitrary length, n, where the drive allele at one locus induces cutting of the wild-type allele at the next locus in the sequence. To describe this mathematically, it is helpful to generalize our notation.

Consider a daisy drive construct with only two loci, as in Section 1.1. We use a "1" bit to denote a wild-type allele, and we use a "0" bit to denote a daisy drive allele. To represent genotypes, we introduce vectors $a = (a_1, a_2)$ and $b = (b_1, b_2)$, where each $a_1, a_2, b_1, b_2 \in \{0, 1\}$. We construct these vectors such that a_1 and b_1 represent the two alleles at the first locus, while a_2 and b_2 represent the two alleles at the second locus. A full genotype is then a list of the two vectors, $[a, b]$. We write the nine possible genotypes for a two-element drive system as:

$$\begin{aligned}
1_{WW}2_{WW} &= [(1,1),(1,1)] \\
1_{WW}2_{WD} &= [(1,1),(1,0)] \\
1_{WW}2_{DD} &= [(1,0),(1,0)] \\
1_{WD}2_{WW} &= [(1,1),(0,1)] \\
1_{WD}2_{WD} &= [(1,1),(0,0)] \\
1_{WD}2_{DD} &= [(1,0),(0,0)] \\
1_{DD}2_{WW} &= [(0,1),(0,1)] \\
1_{DD}2_{WD} &= [(0,1),(0,0)] \\
1_{DD}2_{DD} &= [(0,0),(0,0)]
\end{aligned}$$

Notice that if an individual is heterozygous at a particular locus, then this notation allows for two ways of writing the alleles at that locus. For example, genotype $1_{WD}2_{WD}$ can be written in any one of four equivalent ways: $[(1,1),(0,0)]$, $[(0,0),(1,1)]$, $[(1,0),(0,1)]$, or $[(0,1),(1,0)]$.

When modeling daisy drives with a large number of loci, it is helpful to adopt shorthand notation. To do this, we extend the lengths of a and b to be equal to the number of loci, n. That is, we let $a = (a_1, \dots, a_n)$ and $b = (b_1, \dots, b_n)$, where each $a_i, b_j \in \{0, 1\}$. For example, the genotype $1_{WW}2_{DD}3_{WD}$ can be written $[a, b] = [(1,0,1),(1,0,0)]$ or, equivalently, $[a, b] = [(1,0,0),(1,0,1)]$.

We denote by x_{ab} the frequency of individuals with genotype $[a, b]$. We denote by g_b the rate at which gametes with haplotype b are produced. For an n-element daisy drive, g_b is given by

$$\begin{aligned}
g_b = &\sum_{\alpha,\beta} x_{\alpha\beta} F^{1-\alpha_n\beta_n} \\
&\times \prod_{i=1}^{n} \left\{ \delta_{\alpha_i b_i}\delta_{\beta_i b_i}\left[\delta_{0,b_i} + \alpha_{i-1}\beta_{i-1}\delta_{1,b_i}\right] + (1-\delta_{\alpha_i\beta_i})\left[\frac{\alpha_{i-1}\beta_{i-1}}{2} + (1-\alpha_{i-1}\beta_{i-1})H\delta_{0,b_i}\right]\right\}
\end{aligned} \tag{1}$$

Here, we have defined $\alpha_0 = \beta_0 = 1$. δ_{ij} is the Kronecker delta, defined by $\delta_{ij} = 1$ if $i = j$ and $\delta_{ij} = 0$ if $i \neq j$. In Equations (1), in the sum over α, β when enumerating genotypes, heterozygous loci ($\alpha_i \neq \beta_i$) are each counted once, so there is no double-counting. g_b is linear in each $x_{\alpha\beta}$, where all genotypes $[\alpha, \beta]$ are summed over.

We understand the terms in the factors in brackets as follows. Consider just a single factor in brackets for a particular value of i.

- If $\alpha_i = \beta_i = b_i = 0$, then individuals of genotype $[\alpha, \beta]$ have two identical copies of allele 0 at the ith locus, and those individuals create only gametes with allele 0 at position i.

- If $\alpha_i = \beta_i = b_i = 1$ and $\alpha_{i-1}\beta_{i-1} = 1$, then individuals of genotype $[\alpha, \beta]$ have two identical copies of allele 1 at the ith locus and no copy of allele 0 at the $(i-1)^{\text{th}}$ locus, and those individuals create only gametes with allele 1 at position i.

- If $\alpha_i \neq \beta_i$ and $\alpha_{i-1}\beta_{i-1} = 1$, then individuals of genotype $[\alpha, \beta]$ have a single copy of allele b_i at the ith locus, and without any action from the daisy drive, those individuals create gametes with allele b_i and allele $(1 + (-1)^{b_i})/2$ at position i in equal proportion.

- If $\alpha_i \neq \beta_i$ and $\alpha_{i-1}\beta_{i-1} = 0$, then individuals of genotype $[\alpha, \beta]$ have a single copy of allele b_i at the ith locus, and the daisy drive allele at the $(i-1)^{\text{th}}$ locus cuts the wild-type allele at the ith locus. Homing then occurs with probability H, and gametes with allele 0 at position i are created.

The prefactor $F^{1-\alpha_n\beta_n}$ is the fitness cost associated with the payload. It appears if there is at least one copy of the daisy drive allele at the last position, n, in the daisy chain.

The selection dynamics for an n-element daisy drive are modeled by the following equations:

$$\dot{x}_{ab} = \sum_{\alpha} g_{\alpha} \sum_{\beta} g_{\beta} \prod_{i=1}^{n} [\delta_{a_i b_i}\delta_{\alpha_i a_i}\delta_{\beta_i b_i} + (1 - \delta_{a_i b_i})(1 - \delta_{\alpha_i \beta_i})] - \psi^2 x_{ab} \tag{2}$$

In Equations (2), the haplotypes α and β are summed independently. There is one such equation for each possible genotype $[a, b]$.

We make sense of Equations (2) as follows. Each pair of gametes g_α and g_β makes a new individual.

- If $a_i = b_i = \alpha_i = \beta_i$, then gametes of haplotypes α and β pair to make only individuals with genotype $[a_i, b_i]$ at locus i.

- If $a_i \neq b_i$ and $\alpha_i \neq \beta_i$, then gametes of haplotypes α and β pair to make only individuals with genotype $[a_i, b_i]$ at locus i.

We impose the density constraint

$$\sum_{a,b} x_{ab} = 1 \tag{3}$$

As already noted for Equations (1), in the sum over a, b when enumerating genotypes, heterozygous loci ($a_i \neq b_i$) are each counted once, so there is no double-counting. We use the following identity:

$$\sum_{a,b} \prod_{i=1}^{n} [\delta_{a_i b_i}\delta_{\alpha_i a_i}\delta_{\beta_i b_i} + (1 - \delta_{a_i b_i})(1 - \delta_{\alpha_i \beta_i})] = 1$$

The form of ψ that enforces the density constraint is

$$\psi = \sum_{\alpha} g_{\alpha} \tag{4}$$

2 Evolutionary dynamics of daisy drive resistance

Thus far in this Supplementary Information, we have assumed that there are exactly two alleles at each daisy drive locus: the daisy drive element, D, and the corresponding wild-type, W. However, additional alleles could arise in various ways: standing genetic variation, *de novo* mutation, or

misrepair after cutting could all result in alleles with mismatches between the engineered guide RNAs and their corresponding recognition sequences. Such alleles would be *resistant* to the future effects of daisy-mediated cutting.

Our previous consideration of only two classes of allele was motivated by our presumed biological design: each daisy element was to target a highly conserved essential gene using multiple guide RNAs, and the corresponding daisy drive construct was to contain a genetically recoded copy of the target gene. Under these assumptions, we would expect low rates of standing genetic variation and *de novo* mutation, and targets resulting from misrepair would almost certainly produce nonviable offspring.

However, these assumptions are fairly restrictive. It could be difficult, in practice, to locate highly conserved regions, recode essential genes, and design multiple guide RNAs for every daisy element in a large chain, particularly in time-sensitive situations, such as responding to release of a rogue drive. Thus, in this section, we relax these earlier assumptions by extending our model to account for drive-resistant alleles.

2.1 Two elements

We begin by considering the special case of two daisy drive elements, as in Section 1.1 above. The relevant loci are denoted 1 and 2 as before. Now, however, there are three alleles: the wild-type, W, the drive element, D, and a resistant allele, R, which is immune to the effects of the drive. We assume that resistant alleles primarily arise as the result of misrepair following cutting events (standing genetic variation could be accounted for by simply varying the initial frequency of the R allele). Because only the second locus is acted upon by the drive, we ignore resistance at the first locus.

Now, we consider the case where there is at least one drive element at the first locus (e.g., an individual with genotype 1_{WD} or 1_{DD}). Then there are six cases, depending on the genotype at the second locus:

- WW: The drive element cuts at both W alleles until both are resistant to further cutting. The individual thus converts to genotype 2_{RR} at this locus, and all gametes contain the 2_R allele.

- WD: The drive element cuts at the W allele. Subsequent repair occurs by homologous recombination with probability H, or by nonhomologous end-joining with probability $1 - H$. In the former case, the individual converts to genotype 2_{DD} and all gametes have the 2_D allele. In the latter case, the individual converts to 2_{DR} and produces gametes with 2_D or 2_R alleles with equal proportions.

- WR: The drive element cuts at the W allele. Subsequent repair by either repair pathway results in a resistant allele, so the individual converts to genotype 2_{RR}. Thus, all gametes produced contain the 2_R allele.

- DD: No cutting occurs, so all gametes contain the 2_D allele.

- DR: No cutting occurs, so gametes are produced containing the 2_D or 2_R allele with equal proportions.

- RR: No cutting occurs, so all gametes contain the 2_R allele.

The cases above describe the production probabilities of the various alleles. But what are their effects on fitness? We assume that the payload element, 2_D, confers a dominant fitness cost, c; the

Genotype	1_W2_W	1_W2_D	1_W2_R	1_D2_W	1_D2_D	1_D2_R	Fitness
$1_{WW}2_{WW}$	1	0	0	0	0	0	1
$1_{WW}2_{WD}$	$\frac{1}{2}$	$\frac{1}{2}$	0	0	0	0	F
$1_{WW}2_{WR}$	$\frac{1}{2}$	0	$\frac{1}{2}$	0	0	0	K
$1_{WW}2_{DD}$	0	1	0	0	0	0	F
$1_{WW}2_{DR}$	0	$\frac{1}{2}$	$\frac{1}{2}$	0	0	0	FK
$1_{WW}2_{RR}$	0	0	1	0	0	0	K
$1_{WD}2_{WW}$	0	0	$\frac{1}{2}$	0	0	$\frac{1}{2}$	G
$1_{WD}2_{WD}$	0	$\frac{1+H}{4}$	$\frac{1-H}{4}$	0	$\frac{1+H}{4}$	$\frac{1-H}{4}$	FG
$1_{WD}2_{WR}$	0	0	$\frac{1}{2}$	0	0	$\frac{1}{2}$	GK
$1_{WD}2_{DD}$	0	$\frac{1}{2}$	0	0	$\frac{1}{2}$	0	FG
$1_{WD}2_{DR}$	0	$\frac{1}{4}$	$\frac{1}{4}$	0	$\frac{1}{4}$	$\frac{1}{4}$	FGK
$1_{WD}2_{RR}$	0	0	$\frac{1}{2}$	0	0	$\frac{1}{2}$	GK
$1_{DD}2_{WW}$	0	0	0	0	0	1	G
$1_{DD}2_{WD}$	0	0	0	0	$\frac{1+H}{2}$	$\frac{1-H}{2}$	FG
$1_{DD}2_{WR}$	0	0	0	0	0	1	GK
$1_{DD}2_{DD}$	0	0	0	0	1	0	FG
$1_{DD}2_{DR}$	0	0	0	0	$\frac{1}{2}$	$\frac{1}{2}$	FGK
$1_{DD}2_{RR}$	0	0	0	0	0	1	GK

Table 2: Gamete production probabilities and genotype fitnesses for two-element daisy drive with resistant alleles.

upstream drive element, 1_D, confers a dominant fitness cost, d; and the resistant allele confers a dominant fitness cost, s. We assume that the all-wild-type individual has maximum fitness 1, so that $0 \leq c, d, s \leq 1$. We then define the shorthand notation $F = 1 - c$, $G = 1 - d$, and $K = 1 - s$. These assumptions are summarized in Table 2.

Using these rules, we can formally express the rates at which the six types of gametes are produced in the population. We denote by $g(z)$ the rate (with implicit time-dependence) at which

gametes with haplotype z are produced by individuals in the population.

$$
\begin{aligned}
g(1_W 2_W) &= x(1_{WW} 2_{WW}) + \frac{1}{2} F x(1_{WW} 2_{WD}) + \frac{1}{2} K x(1_{WW} 2_{WR}) \\
g(1_W 2_D) &= \frac{1}{2} F x(1_{WW} 2_{WD}) + F x(1_{WW} 2_{DD}) + \frac{1}{2} F K x(1_{WW} 2_{DR}) + \frac{1+H}{4} F G x(1_{WD} 2_{WD}) \\
&\quad + \frac{1}{2} F G x(1_{WD} 2_{DD}) + \frac{1}{4} F G K x(1_{WD} 2_{DR}) \\
g(1_W 2_R) &= \frac{1}{2} K x(1_{WW} 2_{WR}) + \frac{1}{2} F K x(1_{WW} 2_{DR}) + K x(1_{WW} 2_{RR}) + \frac{1}{2} G x(1_{WD} 2_{WW}) \\
&\quad + \frac{1-H}{4} F G x(1_{WD} 2_{WD}) + \frac{1}{2} G K x(1_{WD} 2_{WR}) + \frac{1}{4} F G K x(1_{WD} 2_{DR}) \\
&\quad + \frac{1}{2} G K x(1_{WD} 2_{RR}) \\
g(1_D 2_W) &= 0 \\
g(1_D 2_D) &= \frac{1+H}{4} F G x(1_{WD} 2_{WD}) + \frac{1}{2} F G x(1_{WD} 2_{DD}) + \frac{1}{4} F G K x(1_{WD} 2_{DR}) \\
&\quad + \frac{1+H}{2} F G x(1_{DD} 2_{WD}) + F G x(1_{DD} 2_{DD}) + \frac{1}{2} F G K x(1_{DD} 2_{DR}) \\
g(1_D 2_R) &= \frac{1}{2} G x(1_{WD} 2_{WW}) + \frac{1-H}{4} F G x(1_{WD} 2_{WD}) + \frac{1}{2} G K x(1_{WD} 2_{WR}) \\
&\quad + \frac{1}{4} F G K x(1_{WD} 2_{DR}) + \frac{1}{2} G K x(1_{WD} 2_{RR}) + G x(1_{DD} 2_{WW}) \\
&\quad + \frac{1-H}{2} F G x(1_{DD} 2_{WD}) + G K x(1_{DD} 2_{WR}) + \frac{1}{2} F G K x(1_{DD} 2_{DR}) \\
&\quad + G K x(1_{DD} 2_{RR})
\end{aligned}
$$

Here, $x(z)$ is the frequency of individuals with genotype z.

The selection dynamics are then modeled by the following system of equations:

$$\begin{aligned}
\dot{x}(1_{WW}2_{WW}) &= g(1_W2_W)^2 - \psi^2 x(1_{WW}2_{WW})\\
\dot{x}(1_{WW}2_{WD}) &= 2g(1_W2_W)g(1_W2_D) - \psi^2 x(1_{WW}2_{WD})\\
\dot{x}(1_{WW}2_{WR}) &= 2g(1_W2_W)g(1_W2_R) - \psi^2 x(1_{WW}2_{WR})\\
\dot{x}(1_{WW}2_{DD}) &= g(1_W2_D)^2 - \psi^2 x(1_{WW}2_{DD})\\
\dot{x}(1_{WW}2_{DR}) &= 2g(1_W2_D)g(1_W2_R) - \psi^2 x(1_{WW}2_{DR})\\
\dot{x}(1_{WW}2_{RR}) &= g(1_W2_R)^2 - \psi^2 x(1_{WW}2_{RR})\\
\dot{x}(1_{WD}2_{WW}) &= 2g(1_W2_W)g(1_D2_W) - \psi^2 x(1_{WD}2_{WW})\\
\dot{x}(1_{WD}2_{WD}) &= 2g(1_W2_D)g(1_D2_W) + 2g(1_W2_W)g(1_D2_D) - \psi^2 x(1_{WD}2_{WD})\\
\dot{x}(1_{WD}2_{WR}) &= 2g(1_W2_R)g(1_D2_W) + 2g(1_W2_W)g(1_D2_R) - \psi^2 x(1_{WD}2_{WR})\\
\dot{x}(1_{WD}2_{DD}) &= 2g(1_W2_D)g(1_D2_D) - \psi^2 x(1_{WD}2_{DD})\\
\dot{x}(1_{WD}2_{DR}) &= 2g(1_W2_D)g(1_D2_R) + 2g(1_W2_R)g(1_D2_D) - \psi^2 x(1_{WD}2_{DR})\\
\dot{x}(1_{WD}2_{RR}) &= 2g(1_W2_R)g(1_D2_R) - \psi^2 x(1_{WD}2_{RR})\\
\dot{x}(1_{DD}2_{WW}) &= g(1_D2_W)^2 - \psi^2 x(1_{DD}2_{WW})\\
\dot{x}(1_{DD}2_{WD}) &= 2g(1_D2_W)g(1_D2_D) - \psi^2 x(1_{DD}2_{WD})\\
\dot{x}(1_{DD}2_{WR}) &= 2g(1_D2_W)g(1_D2_R) - \psi^2 x(1_{DD}2_{WR})\\
\dot{x}(1_{DD}2_{DD}) &= g(1_D2_D)^2 - \psi^2 x(1_{DD}2_{DD})\\
\dot{x}(1_{DD}2_{DR}) &= 2g(1_D2_D)g(1_D2_R) - \psi^2 x(1_{DD}2_{DR})\\
\dot{x}(1_{DD}2_{RR}) &= g(1_D2_R)^2 - \psi^2 x(1_{DD}2_{RR})
\end{aligned}$$

Note that this formulation assumes random mating as before, i.e., that two random gametes come together to form an individual. Also note that products $g(y)g(z)$ represent the pairings of different gametes. At any given time, we require that the total number of individuals sums to one:

$$\sum_z x(z) = 1$$

To enforce this density constraint, we set

$$\psi = g(1_W2_W) + g(1_W2_D) + g(1_W2_R) + g(1_D2_W) + g(1_D2_D) + g(1_D2_R)$$

2.2 Evolutionary dynamics of an n-element daisy drive with resistance

As in Section 1.2 above, we now apply the same concept to a daisy drive chain of arbitrary length, n. To describe this mathematically, we return to and amend our previous notation for an n-element system.

Consider a daisy drive construct with only two loci, as in Section 2.1. We use "W" to denote a wild-type allele, "D" to denote a daisy drive allele, and "R" to denote a resistant allele. To represent genotypes, we introduce vectors $a = (a_1, a_2)$ and $b = (b_1, b_2)$, where each $a_1, a_2, b_1, b_2 \in \{W, D, R\}$. We construct these vectors such that a_1 and b_1 represent the two alleles at the first locus, while a_2 and b_2 represent the two alleles at the second locus. A full genotype is then a list of the two vectors, $[a, b]$.

Below are a few examples of this naming convention applied to the genotypes of the two-element system:

$$1_{WW}2_{WW} = [(W, W), (W, W)]$$
$$1_{WW}2_{WD} = [(W, W), (W, D)]$$
$$1_{WW}2_{DD} = [(W, D), (W, D)]$$
$$1_{WW}2_{DR} = [(W, D), (W, R)]$$
$$1_{WD}2_{WW} = [(W, W), (D, W)]$$
$$1_{WD}2_{WD} = [(W, W), (D, D)]$$

To consider daisy drives of arbitrary length, we extend the lengths of the vectors a and b to be equal to the number of loci, n. That is, we let $a = (a_1, \ldots, a_n)$ and $b = (b_1, \ldots, b_n)$, where each $a_i, b_j \in \{W, D, R\}$. Again, notice that if an individual is heterozygous at a particular locus, then this notation allows for two ways of writing the alleles at that locus. For example, the genotype $1_{DD}2_{RR}3_{DR}$ can be written $[a, b] = [(D, R, D), (D, R, R)]$ or, equivalently, $[a, b] = [(D, R, R), (D, R, D)]$.

We denote by x_{ab} the frequency of individuals with genotype $[a, b]$. We denote by g_b the rate at which gametes with haplotype b are produced. For an n-element daisy drive, g_b is given by

$$g_b = \sum_{\alpha,\beta} x_{\alpha\beta} f(\alpha, \beta) p_{\alpha,\beta}(b) \tag{5}$$

Here we have used shorthand notation: $f(\alpha, \beta)$ is the fitness of an individual with genotype $[\alpha, \beta]$, and $p_{\alpha,\beta}(b)$ is the probability that an individual with genotype $[\alpha, \beta]$ produces a gamete with haplotype b. Notice that this is the same form as our Equations (1) above, with the fitness and gamete production components clearly identified.

The fitness of an $[\alpha, \beta]$ individual, $f(\alpha, \beta)$, is given by:

$$f(\alpha, \beta) = \prod_{i=1}^{n} F_i^{1-(1-\delta_{\alpha_i,D})(1-\delta_{\beta_i,D})} K_i^{1-(1-\delta_{\alpha_i,R})(1-\delta_{\beta_i,R})} \tag{6}$$

Here, $F_i = 1 - c_i$, where c_i is the fitness cost associated with the ith daisy drive element. Similarly, $K_i = 1 - s_i$, where s_i is the fitness cost of resistance at the ith position. δ_{ij} is the Kronecker delta, defined by $\delta_{ij} = 1$ if $i = j$ and $\delta_{ij} = 0$ if $i \neq j$. This formulation assumes dominance of each fitness cost and mutual independence of all costs, as in the two-element system in Section 2.1 above.

Although the above formulation allows us to assign arbitrary costs at each position, we make the following simplifying assumptions in our simulations:

- The cost of resistance at upstream (non-payload) elements is zero: $K_1 = \cdots = K_{n-1} = 1$.
- All upstream (non-payload) drive elements have identical associated fitness costs: $F_1 = \cdots = F_{n-1} = 1 - d$.
- We define a cost, s, associated with resistance to the payload element: $K_n = 1 - s$.
- We define a cost, c, associated with the payload element itself: $F_n = 1 - c$.

Then, the probability, $p_{\alpha,\beta}(b)$, of an $[\alpha,\beta]$ individual producing gamete b is given by:

$$
\begin{aligned}
p_{\alpha,\beta}(b) = \prod_{i=1}^{n} \Bigg\{ & \left(1-\gamma^D_{\alpha_{i-1},\beta_{i-1}}(0)\right) \\
& \times \Big[\delta_{b_i,R}\gamma^W_{\alpha_i,\beta_i}(2) + \delta_{b_i,R}\gamma^R_{\alpha_i,\beta_i}(1)\gamma^W_{\alpha_i,\beta_i}(1) \\
& \quad + \delta_{b_i,R}\gamma^R_{\alpha_i,\beta_i}(2) + \frac{1}{2}\delta_{b_i,R}\gamma^R_{\alpha_i,\beta_i}(1)\gamma^D_{\alpha_i,\beta_i}(1) + \frac{1-H}{2}\delta_{b_i,R}\gamma^W_{\alpha_i,\beta_i}(1)\gamma^D_{\alpha_i,\beta_i}(1) \\
& \quad + \delta_{b_i,D}\gamma^D_{\alpha_i,\beta_i}(2) + \frac{1}{2}\delta_{b_i,D}\gamma^D_{\alpha_i,\beta_i}(1)\gamma^R_{\alpha_i,\beta_i}(1) + \frac{1+H}{2}\delta_{b_i,D}\gamma^D_{\alpha_i,\beta_i}(1)\gamma^W_{\alpha_i,\beta_i}(1)\Big] \\
& + \gamma^D_{\alpha_{i-1},\beta_{i-1}}(0) \\
& \times \Big[\delta_{b_i,W}\gamma^W_{\alpha_i,\beta_i}(2) + \frac{1}{2}\delta_{b_i,W}\gamma^W_{\alpha_i,\beta_i}(1)\gamma^D_{\alpha_i,\beta_i}(1) + \frac{1}{2}\delta_{b_i,W}\gamma^W_{\alpha_i,\beta_i}(1)\gamma^R_{\alpha_i,\beta_i}(1) \\
& \quad + \delta_{b_i,D}\gamma^D_{\alpha_i,\beta_i}(2) + \frac{1}{2}\delta_{b_i,D}\gamma^D_{\alpha_i,\beta_i}(1)\gamma^R_{\alpha_i,\beta_i}(1) + \frac{1}{2}\delta_{b_i,D}\gamma^D_{\alpha_i,\beta_i}(1)\gamma^W_{\alpha_i,\beta_i}(1) \\
& \quad + \delta_{b_i,R}\gamma^R_{\alpha_i,\beta_i}(2) + \frac{1}{2}\delta_{b_i,R}\gamma^R_{\alpha_i,\beta_i}(1)\gamma^W_{\alpha_i,\beta_i}(1) + \frac{1}{2}\delta_{b_i,R}\gamma^R_{\alpha_i,\beta_i}(1)\gamma^D_{\alpha_i,\beta_i}(1)\Big]\Bigg\}
\end{aligned} \tag{7}
$$

Here, we use shorthand notation, $\gamma^c_{\alpha_i,\beta_i}(k)$, to count the number of a particular allele at a particular locus: we define $\gamma^c_{\alpha_i,\beta_i}(k)=1$ if there are k copies ($k=0,1,2$) of allele c ($c\in\{W,D,R\}$) at position i in an individual with genotype $[\alpha,\beta]$. Otherwise, $\gamma^c_{\alpha_i,\beta_i}(k)=0$. This is given by:

$$
\gamma^c_{\alpha_i,\beta_i}(k) = \delta_{k,0}\left[(1-\delta_{\alpha_i,c})(1-\delta_{\beta_i,c})\right] + \delta_{k,1}\left[\delta_{\alpha_i,c}(1-\delta_{\beta_i,c}) + \delta_{\beta_i,c}(1-\delta_{\alpha_i,c})\right] + \delta_{k,2}\left[\delta_{\alpha_i,c}\delta_{\beta_i,c}\right].
$$

For example, $\gamma^W_{\alpha_i,\beta_i}(2)=1$ if there are two copies of a wild-type allele at position i in an $[\alpha,\beta]$ individual; otherwise $\gamma^W_{\alpha_i,\beta_i}(2)=0$. We also define $\alpha_0=\beta_0=W$.

We understand Equations (7) as follows. Inheritance at each locus is independent, so the total probability $p_{\alpha,\beta}(b)$ is the product of inheritance probabilities at each individual position. Consider locus i. There are two possibilities. Either there is a daisy drive allele at the previous locus, which entails $\gamma^D_{\alpha_{i-1},\beta_{i-1}}(0)=0$. (This eliminates the sum in the second pair of square brackets.) Or there is no daisy drive allele at the previous locus, which entails $\gamma^D_{\alpha_{i-1},\beta_{i-1}}(0)=1$. (This eliminates the sum in the first pair of square brackets.)

If there is a daisy drive allele at the previous locus, then the value of the factor in the product of Equations (7) depends on the genotype at the current locus:

- $(\alpha_i,\beta_i)=(W,W)$. This entails $\gamma^W_{\alpha_i,\beta_i}(2)=1$. Only R alleles are produced at locus i. Thus, the factor is 1 if $\delta_{b_i,R}=1$. Otherwise, it is zero.

- $(\alpha_i,\beta_i)=(W,D)$. This entails $\gamma^W_{\alpha_i,\beta_i}(1)\gamma^D_{\alpha_i,\beta_i}(1)=1$. By the action of the drive, D alleles are produced at locus i with probability $(1+H)/2$, or R alleles are produced at locus i with probability $(1-H)/2$. So if $\delta_{b_i,D}=1$, then the factor is $(1+H)/2$. Or if $\delta_{b_i,R}=1$, then the factor is $(1-H)/2$. Otherwise, it is zero.

- $(\alpha_i,\beta_i)=(W,R)$. This entails $\gamma^W_{\alpha_i,\beta_i}(1)\gamma^R_{\alpha_i,\beta_i}(1)=1$. Only R alleles are produced at locus i. Thus, the factor is 1 if $\delta_{b_i,R}=1$. Otherwise, it is zero.

- $(\alpha_i,\beta_i)=(D,D)$. This entails $\gamma^D_{\alpha_i,\beta_i}(2)=1$. Only D alleles are produced at locus i. Thus, the factor is 1 if $\delta_{b_i,D}=1$. Otherwise, it is zero.

- $(\alpha_i, \beta_i) = (D, R)$. This entails $\gamma^D_{\alpha_i,\beta_i}(1)\gamma^R_{\alpha_i,\beta_i}(1) = 1$. Here, D and R alleles are produced at locus i in equal proportions. Thus, the factor is 1/2 if $\delta_{b_i,D} = 1$ or if $\delta_{b_i,R} = 1$. Otherwise, it is zero.

- $(\alpha_i, \beta_i) = (R, R)$. This entails $\gamma^R_{\alpha_i,\beta_i}(2) = 1$. Only R alleles are produced at locus i. Thus, the factor is 1 if $\delta_{b_i,R} = 1$. Otherwise, it is zero.

Similarly, if there is no daisy drive allele at the previous locus, then the value of the factor in the product of Equations (7) depends on the genotype at the current locus. However, because there is no drive, the inheritance probabilities are simply Mendelian:

- $(\alpha_i, \beta_i) = (W, W)$. This entails $\gamma^W_{\alpha_i,\beta_i}(2) = 1$. Only W alleles are produced at locus i. Thus, the factor is 1 if $\delta_{b_i,W} = 1$. Otherwise, it is zero.

- $(\alpha_i, \beta_i) = (W, D)$. This entails $\gamma^W_{\alpha_i,\beta_i}(1)\gamma^D_{\alpha_i,\beta_i}(1) = 1$. There is no drive action, so W alleles and D alleles are produced at locus i in equal proportions. Thus, if $\delta_{b_i,W} = 1$ or $\delta_{b_i,D} = 1$, then the factor is 1/2. Otherwise, it is zero.

- $(\alpha_i, \beta_i) = (W, R)$. This entails $\gamma^W_{\alpha_i,\beta_i}(1)\gamma^R_{\alpha_i,\beta_i}(1) = 1$. Here, W alleles and R alleles are produced at locus i in equal proportions. Thus, if $\delta_{b_i,W} = 1$ or $\delta_{b_i,R} = 1$, then the factor is 1/2. Otherwise, it is zero.

- $(\alpha_i, \beta_i) = (D, D)$. This entails $\gamma^D_{\alpha_i,\beta_i}(2) = 1$. Only D alleles are produced at locus i. Thus, the factor is 1 if $\delta_{b_i,D} = 1$. Otherwise, it is zero.

- $(\alpha_i, \beta_i) = (D, R)$. This entails $\gamma^D_{\alpha_i,\beta_i}(1)\gamma^R_{\alpha_i,\beta_i}(1) = 1$. Here, D alleles and R alleles are produced at locus i in equal proportions. Thus, the factor is 1/2 if $\delta_{b_i,D} = 1$ or $\delta_{b_i,R} = 1$. Otherwise, it is zero.

- $(\alpha_i, \beta_i) = (R, R)$. This entails $\gamma^R_{\alpha_i,\beta_i}(2) = 1$. Only R alleles are produced at locus i. Thus, the factor is 1 if $\delta_{b_i,R} = 1$. Otherwise, it is zero.

The selection dynamics for an n-element daisy drive are then modeled by the following equations:

$$\dot{x}_{ab} = \sum_{\alpha} g_\alpha \sum_{\beta} g_\beta \prod_{i=1}^{n} \Delta^{\alpha_i\beta_i}_{a_ib_i} - \psi^2 x_{ab} \tag{8}$$

Here, as shorthand notation, we define

$$\begin{aligned}\Delta^{\alpha_i\beta_i}_{a_ib_i} = {} & \delta_{a_ib_i}\delta_{\alpha_ia_i}\delta_{\beta_ib_i} \\ & + \gamma^W_{a_i,b_i}(1)\gamma^D_{a_i,b_i}(1)\gamma^W_{\alpha_i,\beta_i}(1)\gamma^D_{\alpha_i,\beta_i}(1) \\ & + \gamma^W_{a_i,b_i}(1)\gamma^R_{a_i,b_i}(1)\gamma^W_{\alpha_i,\beta_i}(1)\gamma^R_{\alpha_i,\beta_i}(1) \\ & + \gamma^D_{a_i,b_i}(1)\gamma^R_{a_i,b_i}(1)\gamma^D_{\alpha_i,\beta_i}(1)\gamma^R_{\alpha_i,\beta_i}(1)\end{aligned}$$

In Equations (8), the haplotypes α and β are summed independently. There is one such equation for each possible genotype $[a, b]$.

We impose the density constraint

$$\sum_{a,b} x_{ab} = 1. \tag{9}$$

We use the following identity:

$$\sum_{a,b}\prod_{i=1}^{n}\Delta_{a_ib_i}^{\alpha_i\beta_i}=1$$

And, as before, the form of ψ that enforces the density constraint is

$$\psi=\sum_{\alpha}g_{\alpha}. \tag{10}$$

2.3 Continuous release

To model a continuous release of individuals carrying the daisy drive construct into a population, we use the following equations:

$$\dot{x}_{ab}=\sum_{\alpha}g_{\alpha}\sum_{\beta}g_{\beta}\prod_{i=1}^{n}\Delta_{a_ib_i}^{\alpha_i\beta_i}+C_{ab}-\left(\psi^2+\sum_{\alpha,\beta}C_{\alpha\beta}\right)x_{ab} \tag{11}$$

A nonzero value of C_{ab} models a flow of individuals of genotype $[a,b]$ into the population. Equations (11) are thus a generalization of Equations (8). ψ is given by Equation (10), and the density constraint, Equation (9), holds at all times.

3 Two-population model for an n-element daisy drive with resistance

We now extend the model from Section 2.3 to include a simple spatial component: two populations connected by gene flow.

3.1 Two-population model without gene flow

First, we consider two populations whose evolutionary dynamics are decoupled. We denote by x_{ab} the frequency of individuals with genotype $[a,b]$ among individuals in the target population, and we denote by y_{ab} the frequency of individuals with genotype $[a,b]$ among individuals in the mainland population. We denote by $g_b^{(T)}$ the rate at which gametes with haplotype b are produced in the target population, and we denote by $g_b^{(M)}$ the same for the mainland population. For an n-element daisy drive, $g_b^{(T)}$ and $g_b^{(M)}$ are given by

$$\begin{aligned}g_b^{(T)}&=\sum_{\alpha,\beta}x_{\alpha\beta}f(\alpha,\beta)p_{\alpha,\beta}(b)\\g_b^{(M)}&=\sum_{\alpha,\beta}y_{\alpha\beta}f(\alpha,\beta)p_{\alpha,\beta}(b)\end{aligned} \tag{12}$$

Here, $f(\alpha,\beta)$ is the fitness of the genotype $[\alpha,\beta]$, and $p_{\alpha,\beta}(b)$ is the probability that an individual of genotype $[\alpha,\beta]$ produces a gamete with haplotype b. These two quantities are given by Equations (6) and (7), respectively.

Equations (12) are essentially identical to Equations (5), except we assume that only individuals in the target population contribute to the target population gamete pool and similarly for the mainland. Thus, the difference between Equations (12) and Equations (5) arises from the separation of the two populations via $g_b^{(T)}$, $g_b^{(M)}$, $x_{\alpha\beta}$, and $y_{\alpha\beta}$.

The selection dynamics for an n-element daisy drive system in two populations are then modeled by the following equations:

$$\dot{x}_{ab} = \sum_{\alpha} g_{\alpha}^{(T)} \sum_{\beta} g_{\beta}^{(T)} \prod_{i=1}^{n} \Delta_{a_i b_i}^{\alpha_i \beta_i} + C_{ab}^{(T)} - \left(\left(\psi^{(T)}\right)^2 + \sum_{\alpha,\beta} C_{\alpha\beta}^{(T)} \right) x_{ab}$$

$$\dot{y}_{ab} = \sum_{\alpha} g_{\alpha}^{(M)} \sum_{\beta} g_{\beta}^{(M)} \prod_{i=1}^{n} \Delta_{a_i b_i}^{\alpha_i \beta_i} + C_{ab}^{(M)} - \left(\left(\psi^{(M)}\right)^2 + \sum_{\alpha,\beta} C_{\alpha\beta}^{(M)} \right) y_{ab}$$

Notice that each population experiences selection dynamics identical to the single-population model given by Equations (11). A nonzero value of $C_{ab}^{(T)}$ models a flow of individuals of genotype $[a, b]$ into the target population, and a nonzero value of $C_{ab}^{(M)}$ models a flow of individuals of genotype $[a, b]$ into the mainland population.

The density constraints are

$$\sum_{a,b} x_{ab} = 1$$

$$\sum_{a,b} y_{ab} = 1$$

To enforce these density constraints, we set

$$\psi^{(T)} = \sum_{\alpha} g_{\alpha}^{(T)}$$

$$\psi^{(M)} = \sum_{\alpha} g_{\alpha}^{(M)}$$

3.2 Two-population model with gene flow

Next, we assume that there is a nonzero rate of migration of individuals from the target population to the mainland population and vice versa. For notational clarity, we define new frequency variables. We denote by X_{ab} (with an uppercase X) the frequency of individuals with genotype $[a, b]$ among individuals in the target population when there is migration, and we denote by Y_{ab} (with an uppercase Y) the frequency of individuals with genotype $[a, b]$ among individuals in the mainland population when there is migration. We denote by $G_b^{(T)}$ (with an uppercase G) the rate at which gametes with haplotype b are produced in the target population when there is migration, and we denote by $G_b^{(M)}$ (with an uppercase G) the same for the mainland population when there is migration. $G_b^{(T)}$ and $G_b^{(M)}$ are given by

$$\begin{aligned} G_b^{(T)} &= \sum_{\alpha,\beta} X_{\alpha\beta} f(\alpha, \beta) p_{\alpha,\beta}(b) \\ G_b^{(M)} &= \sum_{\alpha,\beta} Y_{\alpha\beta} f(\alpha, \beta) p_{\alpha,\beta}(b) \end{aligned} \tag{13}$$

Here, $f(\alpha, \beta)$ is the fitness of the genotype $[\alpha, \beta]$, and $p_{\alpha,\beta}(b)$ is the probability that an individual of genotype $[\alpha, \beta]$ produces a gamete with haplotype b. These two quantities are given by Equations (6) and (7), respectively.

We assume that, over a given time interval, the number of individuals migrating in each direction is equal, so that the population sizes of the target and the mainland each remain constant. The rate

of migration is quantified by the parameter r. We also denote by R the fraction of all individuals that are on the target. (Similarly, $1-R$ is the fraction of all individuals that are on the mainland.) The selection dynamics for an n-element daisy drive system in two populations that are connected by gene flow are then modeled by the following equations:

$$\dot{X}_{ab} = \sum_{\alpha} G_{\alpha}^{(T)} \sum_{\beta} G_{\beta}^{(T)} \prod_{i=1}^{n} \Delta_{a_i b_i}^{\alpha_i \beta_i} + C_{ab}^{(T)} + \frac{r}{R}(Y_{ab} - X_{ab}) - \left(\left(\Psi^{(T)} \right)^2 + \sum_{\alpha,\beta} C_{\alpha\beta}^{(T)} \right) X_{ab}$$

$$\dot{Y}_{ab} = \sum_{\alpha} G_{\alpha}^{(M)} \sum_{\beta} G_{\beta}^{(M)} \prod_{i=1}^{n} \Delta_{a_i b_i}^{\alpha_i \beta_i} + C_{ab}^{(M)} + \frac{r}{1-R}(X_{ab} - Y_{ab}) - \left(\left(\Psi^{(M)} \right)^2 + \sum_{\alpha,\beta} C_{\alpha\beta}^{(M)} \right) Y_{ab} \tag{14}$$

The density constraints are

$$\sum_{a,b} X_{ab} = 1$$

$$\sum_{a,b} Y_{ab} = 1$$

To enforce these density constraints, we set $\Psi^{(T)}$ (with an uppercase Ψ) and $\Psi^{(M)}$ (with an uppercase Ψ) to equal

$$\Psi^{(T)} = \sum_{\alpha} G_{\alpha}^{(T)}$$

$$\Psi^{(M)} = \sum_{\alpha} G_{\alpha}^{(M)}$$

4 N-population model for an n-element daisy drive with resistance

The above treatment is readily extended to a population that consists of N islands. Denote the frequency of individuals of genotype $[a,b]$ on island ℓ (for $1 \leq \ell \leq N$) as $X_{ab}^{(\ell)}$. Gametes with haplotype b are produced on island ℓ at rate $G_b^{(\ell)}$, where $G_b^{(\ell)}$ is given by

$$G_b^{(\ell)} = \sum_{\alpha,\beta} X_{\alpha\beta}^{(\ell)} f(\alpha,\beta) p_{\alpha,\beta}(b)$$

The rate of migration of individuals between islands ℓ and ω is quantified by the parameter $r_{\ell\omega} = r_{\omega\ell}$. The fraction of all individuals in the population that are on island ℓ is denoted by R_ℓ. The dynamics of $X_{ab}^{(\ell)}$ are given by

$$\dot{X}_{ab}^{(\ell)} = \sum_{\alpha} G_{\alpha}^{(\ell)} \sum_{\beta} G_{\beta}^{(\ell)} \prod_{i=1}^{n} \Delta_{a_i b_i}^{\alpha_i \beta_i} + C_{ab}^{(\ell)} + \sum_{\substack{\omega=1 \\ \omega\neq\ell}}^{N} \frac{r_{\ell\omega}}{R_\ell} \left(X_{ab}^{(\omega)} - X_{ab}^{(\ell)} \right) - \left(\left(\Psi^{(\ell)} \right)^2 + \sum_{\alpha,\beta} C_{\alpha\beta}^{(\ell)} \right) X_{ab}^{(\ell)} \tag{15}$$

The density constraints are

$$\sum_{a,b} X_{ab}^{(\ell)} = 1$$

To enforce these density constraints, we set $\Psi^{(\ell)}$ (with an uppercase Ψ) to equal

$$\Psi^{(\ell)} = \sum_{\alpha} G_{\alpha}^{(\ell)}$$

5 Particular case: Daisy-chain versus self-propagating drives on five islands

It is instructive to contrast the evolutionary dynamics of a daisy-chain gene drive with a self-propagating gene drive, where in both cases the evolution occurs in a population consisting of five islands. For simplicity, we assume that individuals are only exchanged between nearby islands, i.e., there is gene flow between islands 1 and 2, between islands 2 and 3, between islands 3 and 4, and between islands 4 and 5. We further assume that these rates of gene flow are all equal, and we assume that each island has the same number of individuals.

In this section, we present the equations necessary to perform simulations of the evolutionary dynamics for each of these scenarios.

5.1 5-population model for an n-element daisy drive

For modeling the dynamics of a daisy-chain gene drive on five islands, we use Equations (15). Substituting $r_{12}/R_1 = r_{21}/R_2 = r_{23}/R_2 = r_{32}/R_3 = r_{34}/R_3 = r_{43}/R_4 = r_{45}/R_4 = r_{54}/R_5 = r$, and setting all other migration rates equal to zero, we obtain

$$\dot{X}_{ab}^{(1)} = \sum_{\alpha} G_{\alpha}^{(1)} \sum_{\beta} G_{\beta}^{(1)} \prod_{i=1}^{n} \Delta_{a_i b_i}^{\alpha_i \beta_i} + C_{ab}^{(1)} + r\left(X_{ab}^{(2)} - X_{ab}^{(1)}\right) - \left(\left(\Psi^{(1)}\right)^2 + \sum_{\alpha,\beta} C_{\alpha\beta}^{(1)}\right) X_{ab}^{(1)}$$

$$\dot{X}_{ab}^{(2)} = \sum_{\alpha} G_{\alpha}^{(2)} \sum_{\beta} G_{\beta}^{(2)} \prod_{i=1}^{n} \Delta_{a_i b_i}^{\alpha_i \beta_i} + C_{ab}^{(2)} + r\left(X_{ab}^{(3)} + X_{ab}^{(1)} - 2X_{ab}^{(2)}\right) - \left(\left(\Psi^{(2)}\right)^2 + \sum_{\alpha,\beta} C_{\alpha\beta}^{(2)}\right) X_{ab}^{(2)}$$

$$\dot{X}_{ab}^{(3)} = \sum_{\alpha} G_{\alpha}^{(3)} \sum_{\beta} G_{\beta}^{(3)} \prod_{i=1}^{n} \Delta_{a_i b_i}^{\alpha_i \beta_i} + C_{ab}^{(3)} + r\left(X_{ab}^{(4)} + X_{ab}^{(2)} - 2X_{ab}^{(3)}\right) - \left(\left(\Psi^{(3)}\right)^2 + \sum_{\alpha,\beta} C_{\alpha\beta}^{(3)}\right) X_{ab}^{(3)}$$

$$\dot{X}_{ab}^{(4)} = \sum_{\alpha} G_{\alpha}^{(4)} \sum_{\beta} G_{\beta}^{(4)} \prod_{i=1}^{n} \Delta_{a_i b_i}^{\alpha_i \beta_i} + C_{ab}^{(4)} + r\left(X_{ab}^{(5)} + X_{ab}^{(3)} - 2X_{ab}^{(4)}\right) - \left(\left(\Psi^{(4)}\right)^2 + \sum_{\alpha,\beta} C_{\alpha\beta}^{(4)}\right) X_{ab}^{(4)}$$

$$\dot{X}_{ab}^{(5)} = \sum_{\alpha} G_{\alpha}^{(5)} \sum_{\beta} G_{\beta}^{(5)} \prod_{i=1}^{n} \Delta_{a_i b_i}^{\alpha_i \beta_i} + C_{ab}^{(5)} + r\left(X_{ab}^{(4)} - X_{ab}^{(5)}\right) - \left(\left(\Psi^{(5)}\right)^2 + \sum_{\alpha,\beta} C_{\alpha\beta}^{(5)}\right) X_{ab}^{(5)}$$

5.2 5-population model for a self-propagating drive

The equations for modeling the dynamics of a self-propagating gene drive on five islands are based on Section S7 of the Supplementary Materials for Noble et al. (2017) (Ref. 1). (For more details and descriptions, please see the writing therein.)

For a self-propagating gene drive, consider that there are $\mathcal{N}$ guide RNAs. There are the drive allele, D, $\mathcal{N}$ "costly" resistant alleles, R_i (with $1 \leq i \leq \mathcal{N}$), $\mathcal{N}$ "neutral" resistant alleles, S_i (with $1 \leq i \leq \mathcal{N}$), and the wild-type allele, S_0.

We use $X_{ab}^{(\ell)}$ to denote the frequency of individuals of genotype $[a, b]$ on island ℓ. The rates at

which each of the $2\mathcal{N}+2$ types of gametes are produced on island ℓ are given by

$$F_D^{(\ell)} = f_{DD}X_{DD}^{(\ell)} + \sum_{k=1}^{\mathcal{N}} p_{R_kD,D} f_{R_kD} X_{R_kD}^{(\ell)} + \sum_{k=0}^{\mathcal{N}} p_{S_kD,D} f_{S_kD} X_{S_kD}^{(\ell)}$$

$$F_{S_i}^{(\ell)} = \sum_{k=0}^{\mathcal{N}} \frac{1+\delta_{ki}}{2} f_{S_kS_i} X_{S_kS_i}^{(\ell)} + \frac{1}{2}\sum_{k=1}^{\mathcal{N}} f_{R_kS_i} X_{R_kS_i}^{(\ell)} + \sum_{k=0}^{i} p_{S_kD,S_i} f_{S_kD} X_{S_kD}^{(\ell)}$$

$$F_{R_i}^{(\ell)} = \sum_{k=1}^{\mathcal{N}} \frac{1+\delta_{ki}}{2} f_{R_kR_i} X_{R_kR_i}^{(\ell)} + \frac{1}{2}\sum_{k=0}^{\mathcal{N}} f_{R_iS_k} X_{R_iS_k}^{(\ell)} + \sum_{k=1}^{i} p_{R_kD,R_i} f_{R_kD} X_{R_kD}^{(\ell)} + \sum_{k=0}^{i-1} p_{S_kD,R_i} f_{S_kD} X_{S_kD}^{(\ell)}$$

From conservation of probability, we have

$$p_{R_kD,D} + \sum_{i=k}^{\mathcal{N}} p_{R_kD,R_i} = 1$$

$$p_{S_kD,D} + \sum_{i=k}^{\mathcal{N}} p_{S_kD,S_i} + \sum_{i=k+1}^{\mathcal{N}} p_{S_kD,R_i} = 1$$

Since type $R_{\mathcal{N}}D$ and type $S_{\mathcal{N}}D$ individuals are fully resistant to being manipulated by the drive construct, they show standard Mendelian segregation in their production of gametes, and we have

$$p_{R_{\mathcal{N}}D,R_{\mathcal{N}}} = p_{S_{\mathcal{N}}D,S_{\mathcal{N}}} = \frac{1}{2}$$

For modeling the dynamics of a self-propagating gene drive on five islands, we use the following equations:

The dynamics of individuals of genotype DD on each island are given by

$$\dot{X}_{DD}^{(1)} = \left(F_D^{(1)}\right)^2 + C_{DD}^{(1)} + r\left(X_{DD}^{(2)} - X_{DD}^{(1)}\right) - \left(\left(\Psi^{(1)}\right)^2 + \sum_{\alpha,\beta} C_{\alpha\beta}^{(1)}\right) X_{DD}^{(1)}$$

$$\dot{X}_{DD}^{(2)} = \left(F_D^{(2)}\right)^2 + C_{DD}^{(2)} + r\left(X_{DD}^{(3)} + X_{DD}^{(1)} - 2X_{DD}^{(2)}\right) - \left(\left(\Psi^{(2)}\right)^2 + \sum_{\alpha,\beta} C_{\alpha\beta}^{(2)}\right) X_{DD}^{(2)}$$

$$\dot{X}_{DD}^{(3)} = \left(F_D^{(3)}\right)^2 + C_{DD}^{(3)} + r\left(X_{DD}^{(4)} + X_{DD}^{(2)} - 2X_{DD}^{(3)}\right) - \left(\left(\Psi^{(3)}\right)^2 + \sum_{\alpha,\beta} C_{\alpha\beta}^{(3)}\right) X_{DD}^{(3)}$$

$$\dot{X}_{DD}^{(4)} = \left(F_D^{(4)}\right)^2 + C_{DD}^{(4)} + r\left(X_{DD}^{(5)} + X_{DD}^{(3)} - 2X_{DD}^{(4)}\right) - \left(\left(\Psi^{(4)}\right)^2 + \sum_{\alpha,\beta} C_{\alpha\beta}^{(4)}\right) X_{DD}^{(4)}$$

$$\dot{X}_{DD}^{(5)} = \left(F_D^{(5)}\right)^2 + C_{DD}^{(5)} + r\left(X_{DD}^{(4)} - X_{DD}^{(5)}\right) - \left(\left(\Psi^{(5)}\right)^2 + \sum_{\alpha,\beta} C_{\alpha\beta}^{(5)}\right) X_{DD}^{(5)}$$

The dynamics of individuals of genotype R_iD on each island are given by

$$\dot{X}^{(1)}_{R_iD} = 2F^{(1)}_{R_i}F^{(1)}_{D} + C^{(1)}_{R_iD} + r\left(X^{(2)}_{R_iD} - X^{(1)}_{R_iD}\right) - \left(\left(\Psi^{(1)}\right)^2 + \sum_{\alpha,\beta} C^{(1)}_{\alpha\beta}\right) X^{(1)}_{R_iD}$$

$$\dot{X}^{(2)}_{R_iD} = 2F^{(2)}_{R_i}F^{(2)}_{D} + C^{(2)}_{R_iD} + r\left(X^{(3)}_{R_iD} + X^{(1)}_{R_iD} - 2X^{(2)}_{R_iD}\right) - \left(\left(\Psi^{(2)}\right)^2 + \sum_{\alpha,\beta} C^{(2)}_{\alpha\beta}\right) X^{(2)}_{R_iD}$$

$$\dot{X}^{(3)}_{R_iD} = 2F^{(3)}_{R_i}F^{(3)}_{D} + C^{(3)}_{R_iD} + r\left(X^{(4)}_{R_iD} + X^{(2)}_{R_iD} - 2X^{(3)}_{R_iD}\right) - \left(\left(\Psi^{(3)}\right)^2 + \sum_{\alpha,\beta} C^{(3)}_{\alpha\beta}\right) X^{(3)}_{R_iD}$$

$$\dot{X}^{(4)}_{R_iD} = 2F^{(4)}_{R_i}F^{(4)}_{D} + C^{(4)}_{R_iD} + r\left(X^{(5)}_{R_iD} + X^{(3)}_{R_iD} - 2X^{(4)}_{R_iD}\right) - \left(\left(\Psi^{(4)}\right)^2 + \sum_{\alpha,\beta} C^{(4)}_{\alpha\beta}\right) X^{(4)}_{R_iD}$$

$$\dot{X}^{(5)}_{R_iD} = 2F^{(5)}_{R_i}F^{(5)}_{D} + C^{(5)}_{R_iD} + r\left(X^{(4)}_{R_iD} - X^{(5)}_{R_iD}\right) - \left(\left(\Psi^{(5)}\right)^2 + \sum_{\alpha,\beta} C^{(5)}_{\alpha\beta}\right) X^{(5)}_{R_iD}$$

The dynamics of individuals of genotype S_iD on each island are given by

$$\dot{X}^{(1)}_{S_iD} = 2F^{(1)}_{S_i}F^{(1)}_{D} + C^{(1)}_{S_iD} + r\left(X^{(2)}_{S_iD} - X^{(1)}_{S_iD}\right) - \left(\left(\Psi^{(1)}\right)^2 + \sum_{\alpha,\beta} C^{(1)}_{\alpha\beta}\right) X^{(1)}_{S_iD}$$

$$\dot{X}^{(2)}_{S_iD} = 2F^{(2)}_{S_i}F^{(2)}_{D} + C^{(2)}_{S_iD} + r\left(X^{(3)}_{S_iD} + X^{(1)}_{S_iD} - 2X^{(2)}_{S_iD}\right) - \left(\left(\Psi^{(2)}\right)^2 + \sum_{\alpha,\beta} C^{(2)}_{\alpha\beta}\right) X^{(2)}_{S_iD}$$

$$\dot{X}^{(3)}_{S_iD} = 2F^{(3)}_{S_i}F^{(3)}_{D} + C^{(3)}_{S_iD} + r\left(X^{(4)}_{S_iD} + X^{(2)}_{S_iD} - 2X^{(3)}_{S_iD}\right) - \left(\left(\Psi^{(3)}\right)^2 + \sum_{\alpha,\beta} C^{(3)}_{\alpha\beta}\right) X^{(3)}_{S_iD}$$

$$\dot{X}^{(4)}_{S_iD} = 2F^{(4)}_{S_i}F^{(4)}_{D} + C^{(4)}_{S_iD} + r\left(X^{(5)}_{S_iD} + X^{(3)}_{S_iD} - 2X^{(4)}_{S_iD}\right) - \left(\left(\Psi^{(4)}\right)^2 + \sum_{\alpha,\beta} C^{(4)}_{\alpha\beta}\right) X^{(4)}_{S_iD}$$

$$\dot{X}^{(5)}_{S_iD} = 2F^{(5)}_{S_i}F^{(5)}_{D} + C^{(5)}_{S_iD} + r\left(X^{(4)}_{S_iD} - X^{(5)}_{S_iD}\right) - \left(\left(\Psi^{(5)}\right)^2 + \sum_{\alpha,\beta} C^{(5)}_{\alpha\beta}\right) X^{(5)}_{S_iD}$$

The dynamics of individuals of genotype R_iS_j on each island are given by

$$\dot{X}^{(1)}_{R_iS_j} = 2F^{(1)}_{R_i}F^{(1)}_{S_j} + C^{(1)}_{R_iS_j} + r\left(X^{(2)}_{R_iS_j} - X^{(1)}_{R_iS_j}\right) - \left(\left(\Psi^{(1)}\right)^2 + \sum_{\alpha,\beta} C^{(1)}_{\alpha\beta}\right) X^{(1)}_{R_iS_j}$$

$$\dot{X}^{(2)}_{R_iS_j} = 2F^{(2)}_{R_i}F^{(2)}_{S_j} + C^{(2)}_{R_iS_j} + r\left(X^{(3)}_{R_iS_j} + X^{(1)}_{R_iS_j} - 2X^{(2)}_{R_iS_j}\right) - \left(\left(\Psi^{(2)}\right)^2 + \sum_{\alpha,\beta} C^{(2)}_{\alpha\beta}\right) X^{(2)}_{R_iS_j}$$

$$\dot{X}^{(3)}_{R_iS_j} = 2F^{(3)}_{R_i}F^{(3)}_{S_j} + C^{(3)}_{R_iS_j} + r\left(X^{(4)}_{R_iS_j} + X^{(2)}_{R_iS_j} - 2X^{(3)}_{R_iS_j}\right) - \left(\left(\Psi^{(3)}\right)^2 + \sum_{\alpha,\beta} C^{(3)}_{\alpha\beta}\right) X^{(3)}_{R_iS_j}$$

$$\dot{X}^{(4)}_{R_iS_j} = 2F^{(4)}_{R_i}F^{(4)}_{S_j} + C^{(4)}_{R_iS_j} + r\left(X^{(5)}_{R_iS_j} + X^{(3)}_{R_iS_j} - 2X^{(4)}_{R_iS_j}\right) - \left(\left(\Psi^{(4)}\right)^2 + \sum_{\alpha,\beta} C^{(4)}_{\alpha\beta}\right) X^{(4)}_{R_iS_j}$$

$$\dot{X}^{(5)}_{R_iS_j} = 2F^{(5)}_{R_i}F^{(5)}_{S_j} + C^{(5)}_{R_iS_j} + r\left(X^{(4)}_{R_iS_j} - X^{(5)}_{R_iS_j}\right) - \left(\left(\Psi^{(5)}\right)^2 + \sum_{\alpha,\beta} C^{(5)}_{\alpha\beta}\right) X^{(5)}_{R_iS_j}$$

The dynamics of individuals of genotype R_iR_j on each island are given by

$$\dot{X}^{(1)}_{R_iR_j} = (2-\delta_{ij})F^{(1)}_{R_i}F^{(1)}_{R_j} + C^{(1)}_{R_iR_j} + r\left(X^{(2)}_{R_iR_j} - X^{(1)}_{R_iR_j}\right) - \left(\left(\Psi^{(1)}\right)^2 + \sum_{\alpha,\beta} C^{(1)}_{\alpha\beta}\right) X^{(1)}_{R_iR_j}$$

$$\dot{X}^{(2)}_{R_iR_j} = (2-\delta_{ij})F^{(2)}_{R_i}F^{(2)}_{R_j} + C^{(2)}_{R_iR_j} + r\left(X^{(3)}_{R_iR_j} + X^{(1)}_{R_iR_j} - 2X^{(2)}_{R_iR_j}\right) - \left(\left(\Psi^{(2)}\right)^2 + \sum_{\alpha,\beta} C^{(2)}_{\alpha\beta}\right) X^{(2)}_{R_iR_j}$$

$$\dot{X}^{(3)}_{R_iR_j} = (2-\delta_{ij})F^{(3)}_{R_i}F^{(3)}_{R_j} + C^{(3)}_{R_iR_j} + r\left(X^{(4)}_{R_iR_j} + X^{(2)}_{R_iR_j} - 2X^{(3)}_{R_iR_j}\right) - \left(\left(\Psi^{(3)}\right)^2 + \sum_{\alpha,\beta} C^{(3)}_{\alpha\beta}\right) X^{(3)}_{R_iR_j}$$

$$\dot{X}^{(4)}_{R_iR_j} = (2-\delta_{ij})F^{(4)}_{R_i}F^{(4)}_{R_j} + C^{(4)}_{R_iR_j} + r\left(X^{(5)}_{R_iR_j} + X^{(3)}_{R_iR_j} - 2X^{(4)}_{R_iR_j}\right) - \left(\left(\Psi^{(4)}\right)^2 + \sum_{\alpha,\beta} C^{(4)}_{\alpha\beta}\right) X^{(4)}_{R_iR_j}$$

$$\dot{X}^{(5)}_{R_iR_j} = (2-\delta_{ij})F^{(5)}_{R_i}F^{(5)}_{R_j} + C^{(5)}_{R_iR_j} + r\left(X^{(4)}_{R_iR_j} - X^{(5)}_{R_iR_j}\right) - \left(\left(\Psi^{(5)}\right)^2 + \sum_{\alpha,\beta} C^{(5)}_{\alpha\beta}\right) X^{(5)}_{R_iR_j}$$

The dynamics of individuals of genotype S_iS_j on each island are given by

$$\dot{X}^{(1)}_{S_iS_j} = (2-\delta_{ij})F^{(1)}_{S_i}F^{(1)}_{S_j} + C^{(1)}_{S_iS_j} + r\left(X^{(2)}_{S_iS_j} - X^{(1)}_{S_iS_j}\right) - \left(\left(\Psi^{(1)}\right)^2 + \sum_{\alpha,\beta} C^{(1)}_{\alpha\beta}\right) X^{(1)}_{S_iS_j}$$

$$\dot{X}^{(2)}_{S_iS_j} = (2-\delta_{ij})F^{(2)}_{S_i}F^{(2)}_{S_j} + C^{(2)}_{S_iS_j} + r\left(X^{(3)}_{S_iS_j} + X^{(1)}_{S_iS_j} - 2X^{(2)}_{S_iS_j}\right) - \left(\left(\Psi^{(2)}\right)^2 + \sum_{\alpha,\beta} C^{(2)}_{\alpha\beta}\right) X^{(2)}_{S_iS_j}$$

$$\dot{X}^{(3)}_{S_iS_j} = (2-\delta_{ij})F^{(3)}_{S_i}F^{(3)}_{S_j} + C^{(3)}_{S_iS_j} + r\left(X^{(4)}_{S_iS_j} + X^{(2)}_{S_iS_j} - 2X^{(3)}_{S_iS_j}\right) - \left(\left(\Psi^{(3)}\right)^2 + \sum_{\alpha,\beta} C^{(3)}_{\alpha\beta}\right) X^{(3)}_{S_iS_j}$$

$$\dot{X}^{(4)}_{S_iS_j} = (2-\delta_{ij})F^{(4)}_{S_i}F^{(4)}_{S_j} + C^{(4)}_{S_iS_j} + r\left(X^{(5)}_{S_iS_j} + X^{(3)}_{S_iS_j} - 2X^{(4)}_{S_iS_j}\right) - \left(\left(\Psi^{(4)}\right)^2 + \sum_{\alpha,\beta} C^{(4)}_{\alpha\beta}\right) X^{(4)}_{S_iS_j}$$

$$\dot{X}^{(5)}_{S_iS_j} = (2-\delta_{ij})F^{(5)}_{S_i}F^{(5)}_{S_j} + C^{(5)}_{S_iS_j} + r\left(X^{(4)}_{S_iS_j} - X^{(5)}_{S_iS_j}\right) - \left(\left(\Psi^{(5)}\right)^2 + \sum_{\alpha,\beta} C^{(5)}_{\alpha\beta}\right) X^{(5)}_{S_iS_j}$$

The density constraints are

$$X^{(\ell)}_{DD} + \sum_{i=1}^{\mathcal{N}} X^{(\ell)}_{R_iD} + \sum_{i=0}^{\mathcal{N}} X^{(\ell)}_{S_iD} + \sum_{i=1}^{\mathcal{N}}\sum_{j=0}^{\mathcal{N}} X^{(\ell)}_{R_iS_j} + \sum_{i=1}^{\mathcal{N}}\sum_{j=1}^{i} X^{(\ell)}_{R_iR_j} + \sum_{i=0}^{\mathcal{N}}\sum_{j=0}^{i} X^{(\ell)}_{S_iS_j} = 1$$

To enforce these density constraints, we set

$$\Psi^{(\ell)} = F^{(\ell)}_D + \sum_{i=1}^{\mathcal{N}} F^{(\ell)}_{R_i} + \sum_{i=0}^{\mathcal{N}} F^{(\ell)}_{S_i}$$

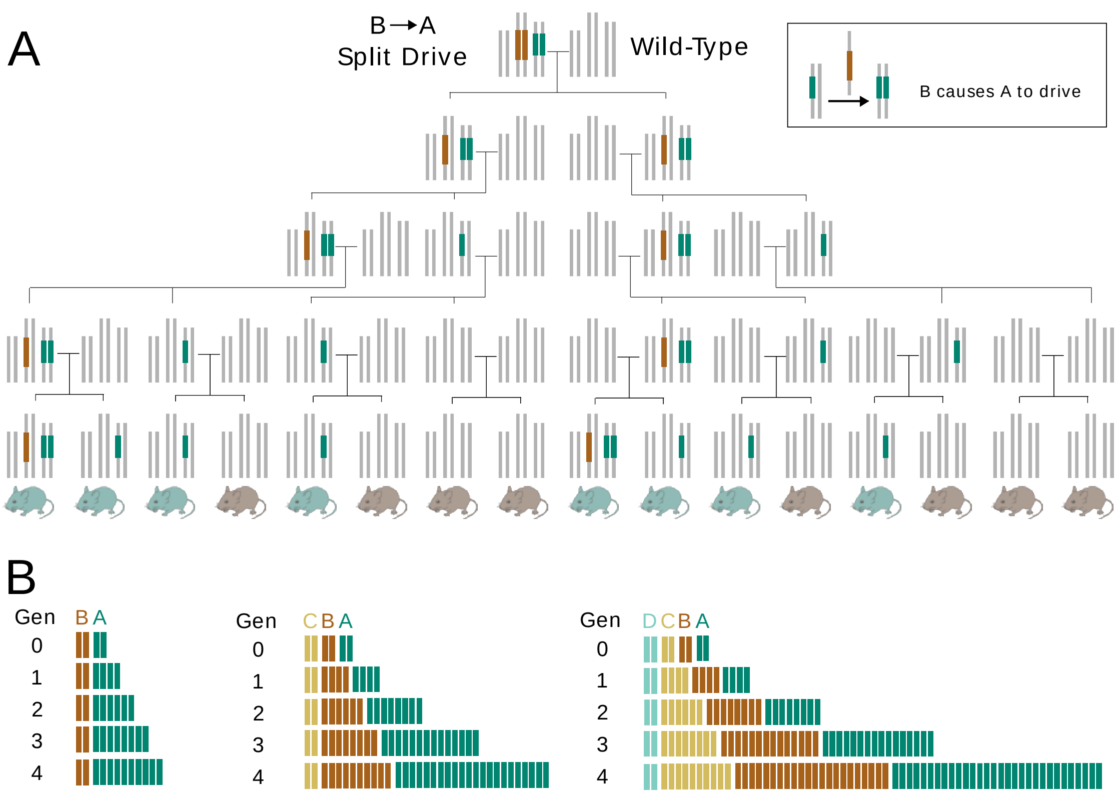

Fig. S1. B→A "split" drives and daisy drive family tree analysis. (*A*) Family tree resulting from a single-organism release of a B→A split drive in a large wild-type population in the absence of selection. (In reality, B elements would be deleterious and thus decline in frequency over time.) For comparison, a C→B→A daisy drive is shown in main text Fig. 1C. Green mice have at least one copy of the cargo A element, while grey mice have only the wild-type allele at that locus. (*B*) A graphical depiction of total alleles in a population per generation for B→A through D→C→B→A daisy drives. Throughout, chromosome illustrations represent genotypes of germline cells after drive has occurred.

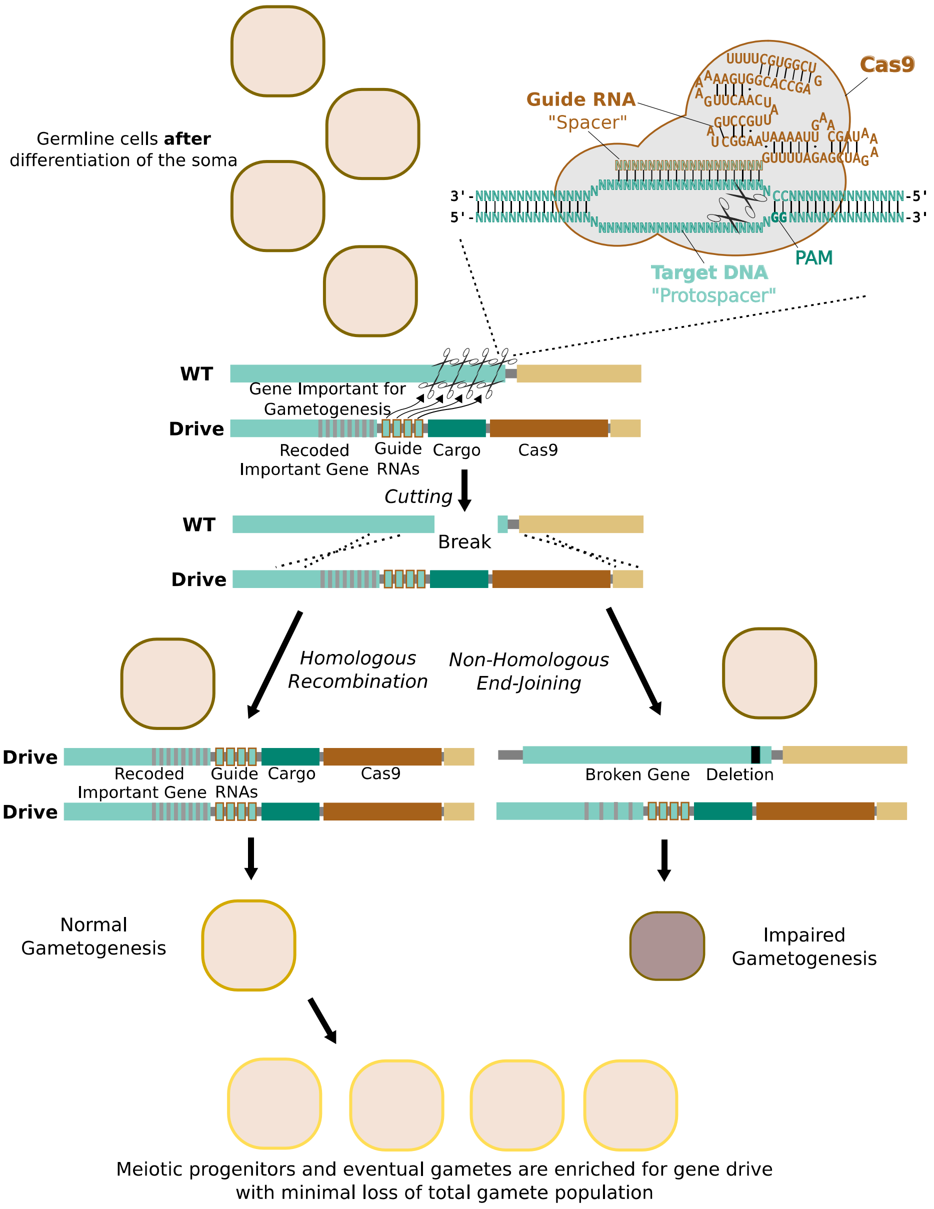

Fig. S2. A potential means of reducing the fitness cost resulting from incorrect repair. One strategy might involve targeting a gene whose loss impairs gametogenesis, such as a ribosomal gene. Increased replication of correctly repaired cells carrying the drive system could potentially result in a wild-type number of gametes, all of which carry the drive system.

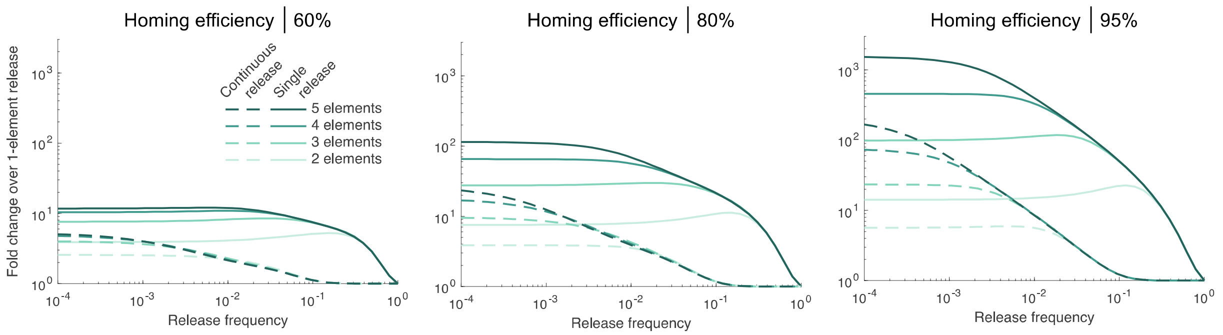

Fig. S3. Fold-change in daisy drive cargo frequency after 20 generations for various daisy chain lengths relative to a release of organisms only containing the cargo. (left) Homing efficiency is assumed to be 60%, and (middle) 80% and (right) 95%. All figures assume 10% cargo fitness cost, 0.01% upstream element cost and neutral resistance. Solid lines correspond to a single release with initial release frequency indicated by the horizontal axes, while dashed lines correspond to continuous releases with frequency indicated by the horizontal axes. See SI Text Section 2.3 for details on our continuous release implementation.

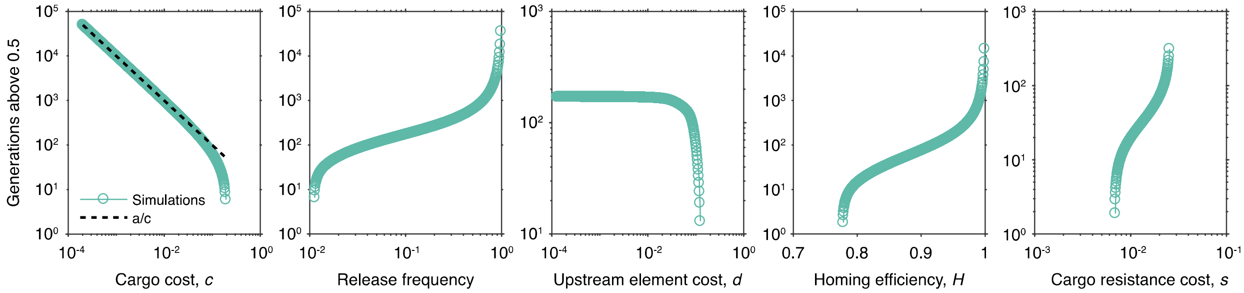

Fig. S4. Analysis of the time that a 3-element (CBA) daisy-chain cargo element remains above 50% frequency in a single population. In each plot, the parameter indicated on the horizontal axis is varied, and the other parameters are fixed. The fixed values in the first four panels are: cargo element (A) fitness cost, *c*, 5%, release frequency 10%, upstream element (C, B) fitness cost, *d*, 0.01%, homing efficiency, *H*, 95%, cargo resistance cost, *s*, 0. In the far-right panel, all parameters are identical except the release frequency is 1%. In the first panel, we additionally plot a function a/c, fitted with *a*=9.99, to illustrate the inverse relationship between cargo time above 50% and cargo fitness cost, *c*, when *c* is low. The model used throughout is described in SI Text Section 2.2.

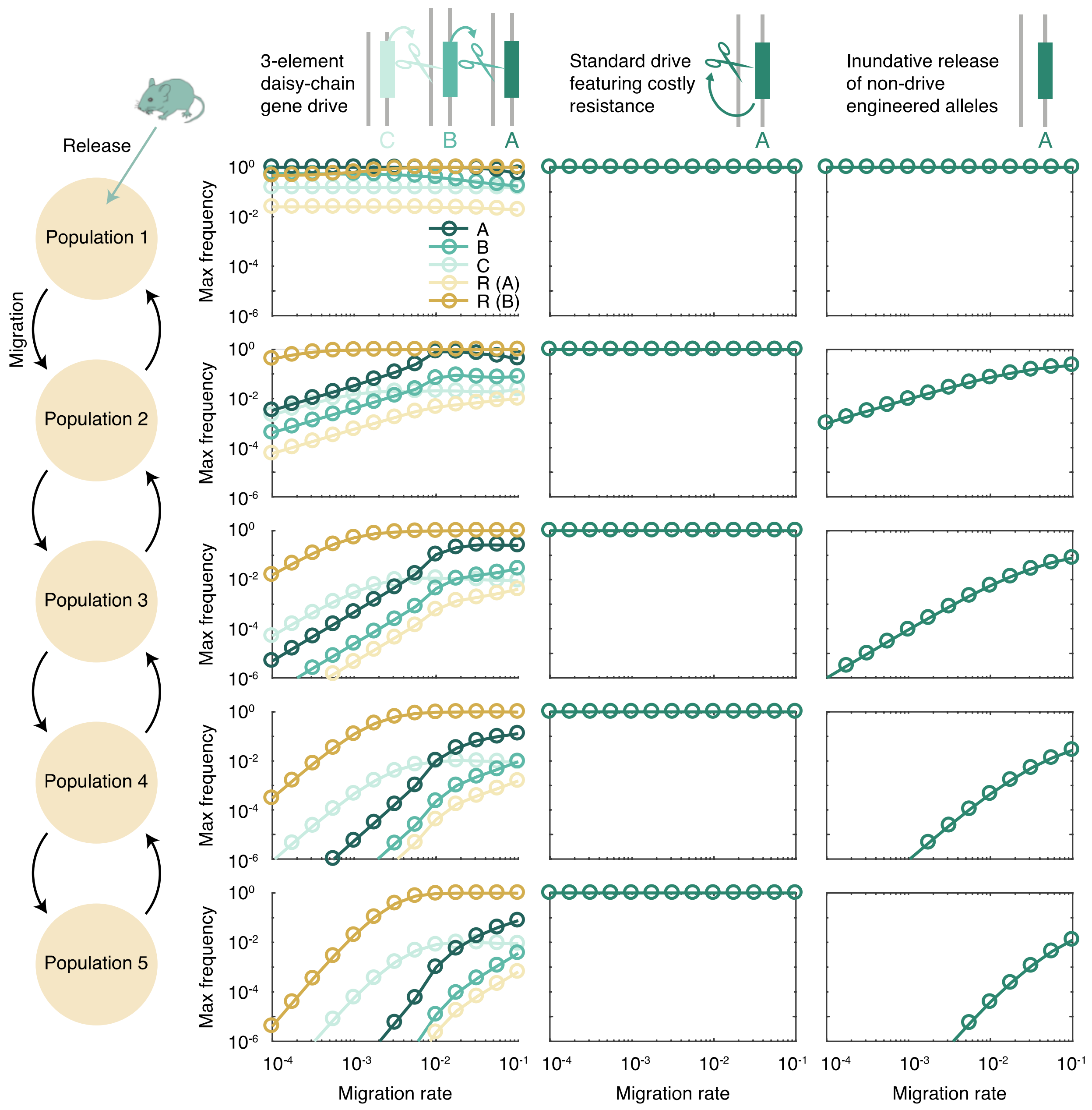

Fig. S5. Further analysis of the 5-population model, shown in Fig. 4 of the main text and described in SI Text Section 5. Three drive systems are considered, as in main text Fig. 4: CBA daisy-chain drive (left), self-propagating drive (middle) and inundative non-drive release (right). We assume 5 equally-sized populations connected in a chain via one constant migration rate, which varies on the horizontal axes from 10^{-4} to 10^{-1}. (e.g., 10^{-2} corresponds to a scenario where each population is connected to its neighbors via a migration rate of 10^{-2} in each direction.) Maximum frequencies of each allele in each population over 500 generations are then plotted as functions of the migration rate.

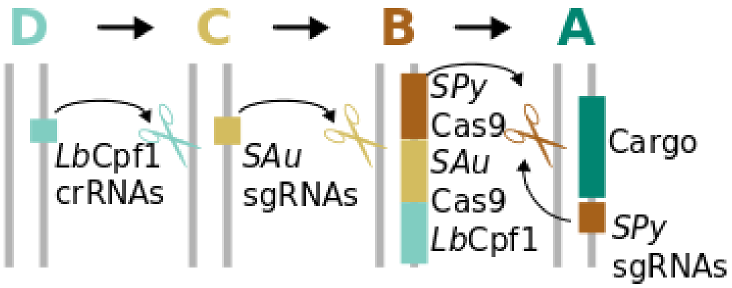

Fig. S6. Daisy drive systems can be constructed using orthogonal Cas9 elements. Such a drive system is resistant to conversion into a daisy necklace, which would require a recombination event that moved the entire Cas9 gene and associated guide RNAs into a subsequent locus in the daisy-chain. Ensuring that all the Cas9 proteins are expressed appropriately without re-using promoters and thereby creating homology between elements could be challenging.

```
template         1 -----------------------------------------N----NNC-AAGTTVVVATAAGGC--------------N
Br_S.pyogenes    1 -----------------TTGTTGGAACCATTCAAAACAGCAT----AGC-AAGTTAAAATAAGGC--------------T
Br_S.dysagalact  1 -----------------TTGTTGGAACCATTCAAAACAACGT----AGC-AAGTTAAAATAAGGC--------------T
Br_S.equi        1 -----------------CCTATGGAACTATTCAATACAGCAT----AGCAAAGTTAAAATAAGGC-------------TT
Br_S.thermophil  1 ----------------CTTACACAGTTACTTAAATCTTGCAG----AAG-CTACAAAGATAAGGC-------------TT
Br_S.salivarius  1 -----------------GGTTTGAAACCATTCGAAACAATAC----AGCAAAGTTAAAATAAGGC-------------TT
Br_S.gallolytic  1 -----------------TTGTTGGAGCTATTCGAAACAACAC----AGC-GAGTTAAAATAAGGC-------------TT
Br_S.lutetiensi  1 -----------------TTGTTGGAACTATTCGAAACAACAC----AGT-GAGTTAAAATAAGGC-------------TT
Br_S.anginosisB  1 -----------------ATGTTGGAATCATTCGAAACAACAC----AGC-AAGTTAAAATAAGGC-------------TT
Br_S.mitis       1 -----------------TCGTTGGAACTATTCGAAACAACAC----AGCAAAGTTAAAATAAGGC-------------TT
Br_S.sanguinis   1 -----------------TTGTTGGAACTATTCGAAACAACAC----AGC-AAGTTAAAATAAGGC-------------TT
Br_S.oralis      1 -----------------TTGTTGGAACTATTCGAAACAACAC----AGC-AAGTTAAAATAAGGC-------------TT
Br_S.mutans      1 ----------------CTTACACAGTTACTTAAATCTTGCAG----AAG-CTACAAAGATAAGGC-------------TT
Br_S.intermediu  1 ----------------CTTACACAGTTACTTAAATCTTGCAG----AAG-CTACAAAGATAAGGC-------------TT
Br_S.anginosusA  1 ----------------CTTACACAGTTACTTAAATCTTGCAG----AAG-CTACAAAGATAAGGC-------------TT
Br_S.thermophil  1 ----------------CTTACACAGTTACTTAAATCTTGCAG----AAG-CTACAAAGATAAGGC-------------TT
Br_S.vestibular  1 ----------------CTTACACAGTTACTTAAATCTTGCAG----AAG-CTACAAAGATAAGGC-------------TT
Br_S.gordonii    1 ----------------CTTACACAGTTACTTAAATCTTGCAG----AAG-CTACAAAGATAAGGC-------------TT
Br_S.parasangui  1 ----------------CTTACACAGTTACTTAAATCTTGCAG----AAG-CTACAAAGATAAGGC-------------TT
Br_S.orisratti   1 ----------------CTTGCACAGTTACTTAAATCTTGCAG----AGC-CTACAAAGATAAGGC-------------TT
Br_S.henryi      1 ----------------CTTGCACAGTTACTTAAATCTTGCTG----AGC-CTACAAAGATAAGGC-------------TT
Br_S.infantariu  1 ----------------CTTGCACGGTTACTTAAATCTTGCAG----AGC-CTACAAAGATAAGGC-------------TT
Ch_C.jejuni      1 ------------------------------AAGAAATTTAAA----AAG-GGACTAAAATAAAGAGTT--TGCGGGACTC
Ch_F.novicida    1 ----------------------------ATCTAAAATTATAA--ATGTA-CCAAATAATTAATGC-------------TC
Ch_S.thermophil  1 ------TTG-------TGGTTTGAAACCATTCGAAACAACAC----AGC-GAGTTAAAATAAGGC-------------TT
Ch_M.mobile      1 TGTATTTCGAAATACAGATGTACAGTTAAGAATACATAAGAATGATACA-TCACTAAAAAAAGGC-------------TT
Ch_L.innocua     1 -------------------ATTGTTAGTATTCAAAATAACAT----AGC-AAGTTAAAATAAGGC-------------TT
Ch_S.pyogenes    1 -------------------GTTGGAACCATTCAAAACAGCAT----AGC-AAGTTAAAATAAGGC--------------T
Ch_S.mutans      1 -------------------GTTGGAATCATTCGAAACAACAC----AGC-AAGTTAAAATAAGGCAGTGATTTTTAATCC
Ch_S.thermophil  1 ------TTG-------TGGTTTGAAACCATTCGAAACAACAC----AGC-GAGTTAAAATAAGGC-------------TT
Ch_N.meningitid  1 ---------------ACATATTGTCGCACTGCGAAATGAGAA----CCG-TTGCTACAATAAGGCC--------------
Ch_P.multocida   1 ---------------GCATATTGTTGCACTGCGAAATGAGAG----ACG-TTGCTACAATAAGGC---------------

template        21 AGTCCGTYHYCANNNNGRRA--NNNNG-GCACCGAKTCGGTGC-------------------------------------
Br_S.pyogenes   45 AGTCCGTTATCAACTTGAAA--AAGTG-GCACCGAGTCGGTGCTTTTTTT------------------------------
Br_S.dysagalact 45 AGTCCGTTATCAACTTGAAA--AAGTG-GCACCGAGTCGGTGCTTTTT--------------------------------
Br_S.equi       47 TGTCCGTAATCAACCTGAAA--AGGGGAGCACCGAATCGGTGCTTTTTT-------------------------------
Br_S.thermophil 47 AGTCCGTACTCAACTTGAAA--AGGTG-GCACCGATTCGGAAGGC-------------TTCATGCCGAAATCAACACCCT
Br_S.salivarius 47 AGTCCGTATTCAACTTGAGA--AAGTG-GCACCGATTCGGTGCTTTTTT-------------------------------
Br_S.gallolytic 46 TGTCCGTACACAACTTGTAA--AAGTGGCACCCGATTCGGGTGCGTTTTTTT----------------------------
Br_S.lutetiensi 46 TGTCCGTACACAACTTATAA--AAGTGGCACCCGATTCGGATGCATTTTTT-----------------------------
Br_S.anginosisB 46 TGTCCGTACTCAACTT-AAA--AAGTGCGCACCGATTCGGTGCTTTTTT-------------------------------
Br_S.mitis      47 TGTCCGTACACAACTTGAAA--AAGTGCGCACCGATTCGGTGCTTTTTT-------------------------------
Br_S.sanguinis  46 TGTCCGTACACAACTTGAAA--AAGTGCGCACCGATTCGGTGCTTTTTT-------------------------------
Br_S.oralis     46 TGTCCGTACACAACTTGAAA--AAGTGCGCACCGATTCGGTGCTTTTT--------------------------------
Br_S.mutans     47 CATGCCGAAATCAACACCCT--ATCTATTATAAGATAGGGTGTTTT----------------------------------
Br_S.intermediu 47 CATGCCGAAATCAACACCCT--GTC-----TATGACGGGGTGTTTT----------------------------------
Br_S.anginosusA 47 CATGCCGAAATCAACACCCT--GTC-----TATGACGGGGTGTTTT----------------------------------
Br_S.thermophil 47 AGTCCGTACTCAACTTGAAA--AGGTG-GCACCGATTCGGAAGGC-------------TTCATGCCGAAATCAACACCCT
Br_S.vestibular 47 CATGCCGAAATCAACACCCT--GTCA-TTTTATGGCAGGGTGTTTT----------------------------------
Br_S.gordonii   47 CATGCCGAATTCAACACCCT--GTCA--TTTATGGCGGGGTGTTTT----------------------------------
Br_S.parasangui 47 CATGCCGAATTCAACACCCT--GTCA--TTTATGGCGGGGTGTTTT----------------------------------
Br_S.orisratti  47 TATGCCGAAATCAAGCACCC--C-----GTTTATACGAGGTGCTTTT---------------------------------
Br_S.henryi     47 CATGCCGAAATCAAGCACCC--CCGT-TTTTAACGAGGGGTGCTTTT---------------------------------
Br_S.infantariu 47 CATGCCGAATTCAAGCACCC--CA---TGTTTACATGGGGTGCTTTT---------------------------------
Ch_C.jejuni     44 TGCGGGGTTACAATCCCCTA--AAAC--------------CGCTTTT---------------------------------
Ch_F.novicida   37 TGTAATCATTTAAAAGTATTTTGAACGGACCTCTGTTTGACACGTCTGAATAACTAAAAA--------------------
Ch_S.thermophil 50 CATGCCGAAATCAACACCCT--GTCA-TTTTATGGCAGGGAAGGC-------------TTAGTCCGTACTCAACTTGAAA
Ch_M.mobile     67 TATGCCGTAACTACTACTTA-------TTTTCAAAATAAGTAGTTTTTTTT-----------------------------
Ch_L.innocua    44 TGTCCGTTATCAACTTTTAATTAAGTA-GCGCTGTTTCGGCGCTTTTTTT------------------------------
Ch_S.pyogenes   43 AGTCCGTTATCAACTTGAAA--AAGTG-GCACCGAGTCGGTGCTTTTTTT------------------------------
Ch_S.mutans     57 AGTCCGTACACAACTTGAAA--AAGTGCGCACCGATTCGGTGCTTTTTTATTT---------------------------
Ch_S.thermophil 50 CATGCCGAAATCAACACCCT--GTCA-TTTTATGGCAGGGAAGGC-------------TTAGTCCGTACTCAACTTGAAA
Ch_N.meningitid 47 ------------GTCTGAAA--AGATGTGCCGCAACGCTCTGCCCCTTAAAGCTTCTGCTTTAAGGGG---------CA-
Ch_P.multocida  46 ------------TTCTGAAA--AGAATGACCGTAACGCTCTGCCCCTTGTGATTCTTAATTGCAAGGGGCATCGTTTTT-
```

Fig. S7. Multiple sequence alignment of existing tracrRNA sequences from closely related Cas9 systems with the tracrRNA component of our sgRNA template (i.e., the template from Fig. 5C, GTNNNAGAGNNN--GRRA--NNNCAAGTTVVVATAAGGCNAGTCCGTYHYCANNNN-GRR-A-NNNNGGCACCGAKTCGGTGC). The sequences with names beginning "Br_" are taken from Fig. S2 of Briner et al., *Mol. Cell* (2014) (Ref. 36 in the main text), and the sequences with names beginning "Ch_" are taken from Fig. 4 of Chylinski et al., *RNA Biol.*, (2013) (Ref. 45 in the main text). Alignments were performed via the MAFFT program with default parameters (Refs. (46, 47) from the main text). Note that mismatches between the template above and the template shown in Fig. 5B-C were empirically tested and found to be tolerated.

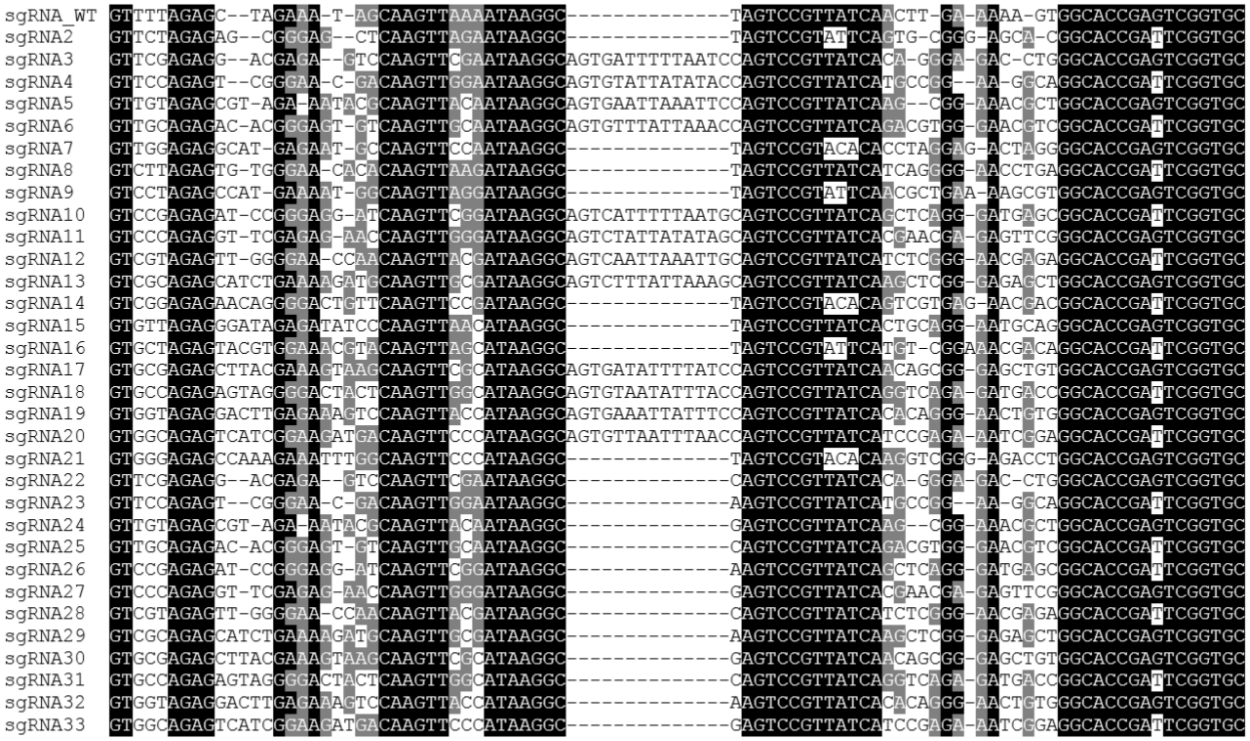

Fig. S8. Complete list of sequence-divergent guide RNAs generated and assayed using the transcriptional activation reporter.

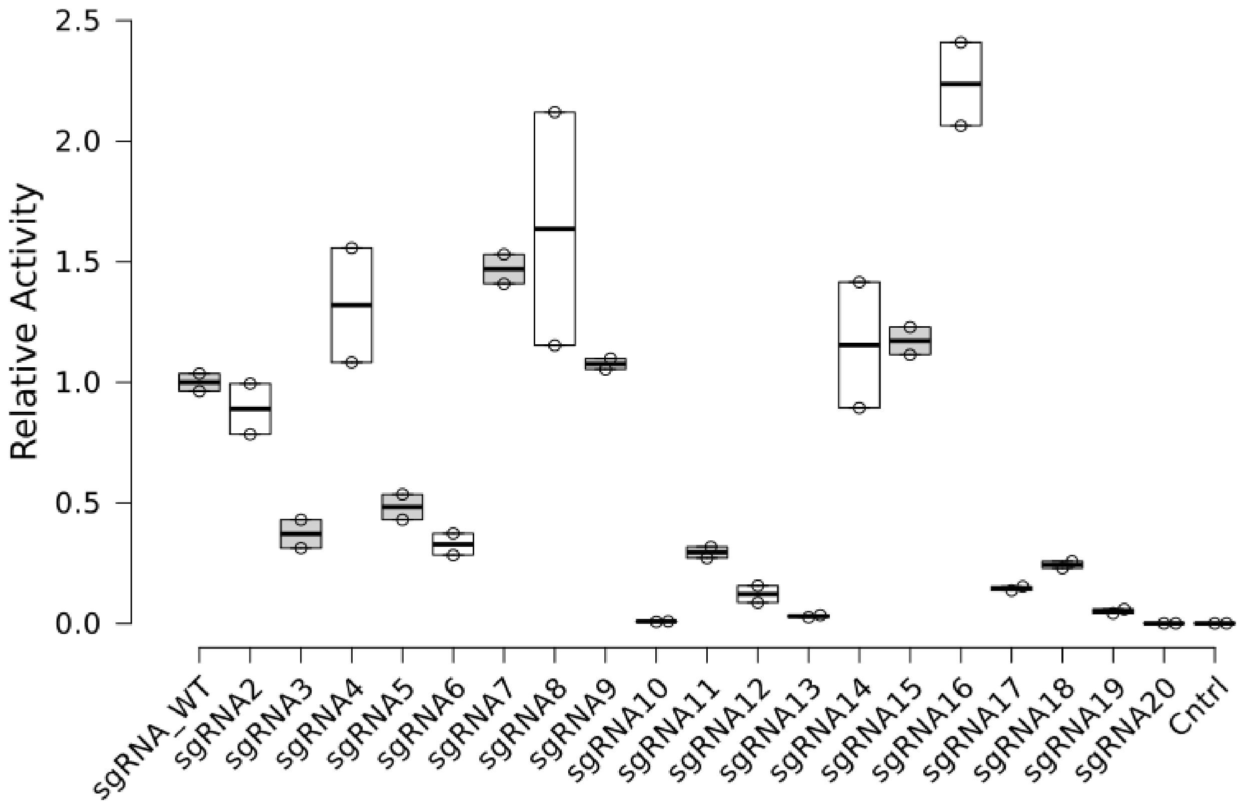

Fig. S9. Results of the pilot screen of the first set of designed sgRNA sequences. #3-6, #10-13, and #17-20 all carried the extra insert; the latter 8 displayed markedly lower activity and were not further considered. The cause of the difference is unclear, although it is worth noting that these all had longer stem-loops than did #3-6, all of which were closer to the activity of the standard or 'wild-type' sgRNA.

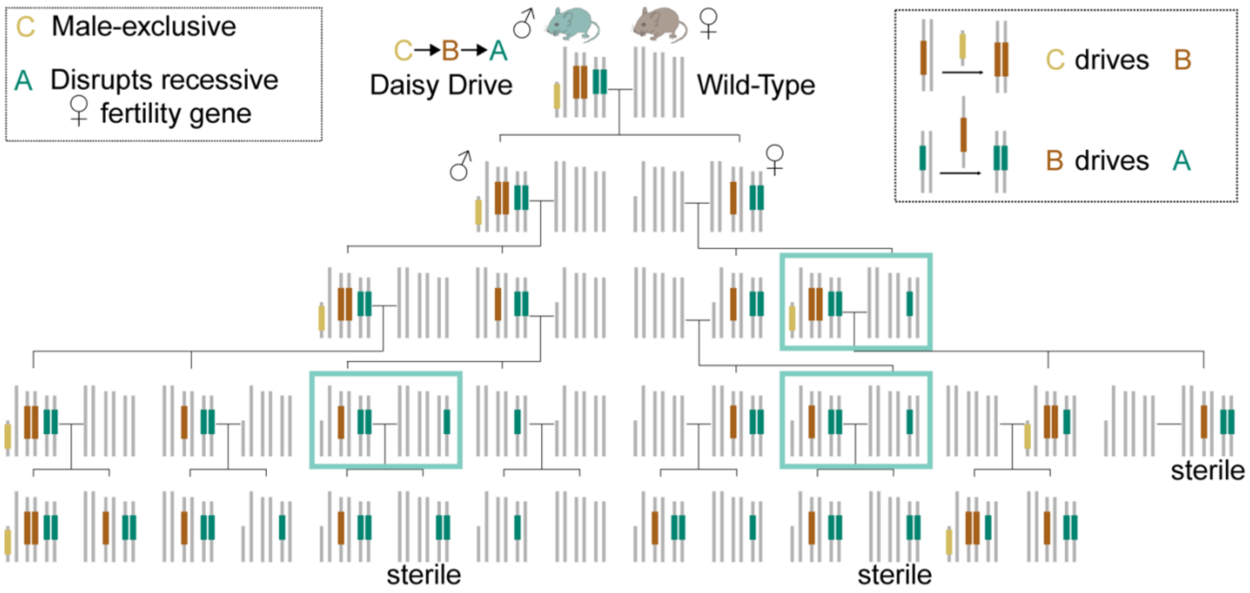

Fig. S10. Potential family tree of a C→B→A genetic load daisy drive for which the cargo in the A element disrupts a female fertility gene. The C element is male-linked, ensuring that it does not suffer a fitness cost from the loss of female fertility. Mating events between two parents carrying the A element (boxed) can produce sterile female offspring that will suppress the population. Males do not suffer a fitness cost due to disruption of female-specific fertility genes. Genome illustrations depict germline cells after drive has occurred. Females are placed on the right side in each pair of individuals.

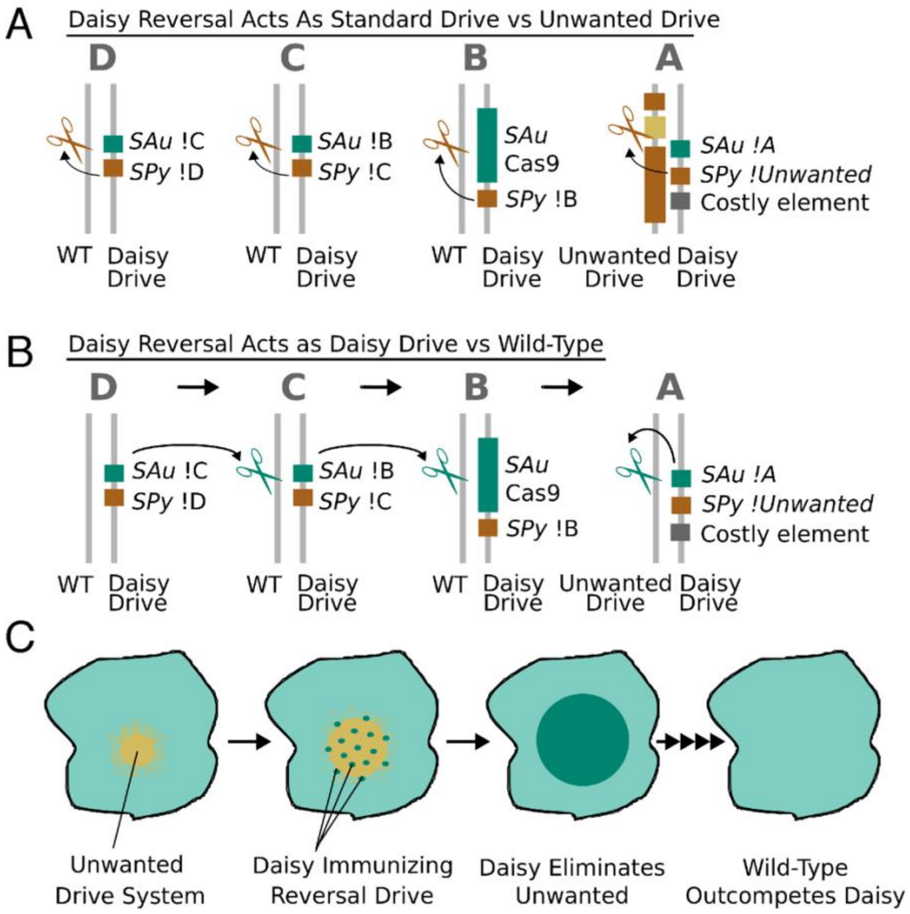

Fig. S11. Utility of a costly daisy reversal drive with orthogonal Cas9 elements in achieving complete genetic reversal of an unwanted drive system to wild-type. Suppose an unwanted drive system has spread a harmful cargo (yellow) through the target locus A via the commonly used Cas9 protein from *S. pyogenes*. (*A*) A daisy reversal drive system uses guide RNAs for *S. pyogenes* Cas9 to copy all elements while overwriting the unwanted drive system and its cargo. (*B*) The same daisy reversal drive system spreads as a normal daisy drive using its own orthogonal CRISPR system (e.g. *S. aureus* Cas9) on encountering wild-type sequences. (*C*) An unwanted drive system is countered by releasing the daisy reversal system at multiple sites. The daisy drive system efficiently overwrites the unwanted drive system throughout its range, spreading into and through the wild-type sequences at the edges of that range to ensure that it reaches and eliminates every copy. This immediately eliminates the harmful cargo. Because the A element of the daisy drive system is costly and the other elements are always co-resident with it due to the daisy drive effect, all elements of the daisy drive will be outcompeted and eliminated by wild-type alleles over time, potentially leading to complete genetic reversal.

References

1. Noble, C., Olejarz, J., Esvelt, K. M., Church, G. M. & Nowak, M. A. Evolutionary dynamics of CRISPR gene drives. *Sci. Adv.* **3,** (2017).
